# Supplementary material for: V-Cornea: A computational model of corneal epithelium homeostasis, injury, and recovery
Source: PLoS Comput Biol. 2025 Dec 26;21(12):e1013410. doi: 10.1371/journal.pcbi.1013410 (PMC12768419; doi:10.1371/journal.pcbi.1013410)
Supplement: S1 Text — Detailed description of the equations governing EGF-dependent growth, density-dependent inhibition (Hill functions), and the volume-threshold mitosis rules used to control cell proliferation. (DOCX) [file pcbi.1013410.s001.docx]

S1 Text. V‑Cornea Supplemental Mathematical Formulation for Growth and Mitosis.
Manuscript Title: V-Cornea: A computational model of corneal epithelium homeostasis, injury, and recovery
Authors: Joel Vanin ^a^, Michael Getz ^a^, Catherine Mahony ^b^, Thomas B. Knudsen ^a^ & James A. Glazier ^a*^
Affiliations: ^a^ Department of Intelligent Systems Engineering and Biocomplexity Institute, Indiana University, Bloomington, Indiana, United States of America; ^b^ Procter & Gamble Technical Centre, Reading, United Kingdom;

# S1. Growth Dynamics Mathematical Formulation

## S1.1 EGF-Dependent Growth (Hill Function)

For basal and stem cell types, the growth factor due to EGF concentration is defined as:

$$\begin{aligned} {G_{n}}_{EGF,i}=\left( \frac{{EG{F_{n}}_{,i}}^{4}}{{{k_{m}}_{EGF,i}}^{4}+{EG{F_{n}}_{i}}^{4}} \right),\#\left( S1 \right) \end{aligned}$$

where $EG{F_{n}}_{i}$ is the average EGF concentration in cell *i* at time *n*. We define:

$$\begin{aligned} EG{F_{n}}_{i} = \frac{{\vartheta_{n}}_{EGF,i}}{{V_{n}}_{i}} ,\#\left( S2 \right) \end{aligned}$$

where

$$\begin{aligned} {\vartheta_{n}}_{EGF,i} = \sum_{p\in i} F_{EGF}\left( p \right),\#\left( S3 \right) \end{aligned}$$

i.e.,${\vartheta_{n}}_{EGF,i}$​ is the total EGF amount inside cell *i*, obtained by summing the EGF field $F_{EGF}$ over all lattice pixels p belonging to cell *i*. ${V_{n}}_{i}$​ is the cell’s volume (in number of pixels or voxels) at time *n*. The parameter ${k_{m}}_{EGF,i}$ is the half‐maximal EGF concentration for cell *i*.

## S1.2 Density-Dependent Growth Inhibition (Hill Function)

Cells experience density-dependent growth inhibition described by another Hill‐type function:

$$\begin{aligned} {G_{n}}_{density,i}=\left( \frac{{k_{m}}_{density,i}{}^{4}}{{k_{m}}_{density,i}{}^{4}+{{P_{n}}_{i}}^{4}} \right) \# \end{aligned}\left( S4 \right)$$

where ${P_{n}}_{i}$​ is the effective “pressure” inside cell *i* at time *n*, derived from its volume deviation (see below). The parameter ${k_{m}}_{density,i}$ is the half‐maximal pressure for growth inhibition.

## S1.3 Effective Pressure Calculation

The volume energy term in the Hamiltonian is:

$$\begin{aligned} H_{volume,i}=\lambda_{v,i}\left( V_{i}-V_{target,i} \right)^{2} \#\left( S5 \right) \end{aligned}$$

where $\boldsymbol{\lambda}_{\boldsymbol{v,i}}$**​** is the volume constraint parameter for cell $i$; $\boldsymbol{V}_{\boldsymbol{i}}$**​** is the actual (current) volume of the cell $\boldsymbol{i}$**,** and $\boldsymbol{V}_{target\boldsymbol{,i}}$ is the target volume of the cell $\boldsymbol{i}$.

The effective pressure inside cell *i* is then:

$$\begin{aligned} {P_{n}}_{i}=\frac{\partial H_{volume,i}}{\partial V_{i}}=2\lambda_{v,i}\left( c-V_{target,i} \right)\#\left( S6 \right) \end{aligned}$$

this pressure term reflects how far the cell’s volume is from its target.

## S1.4 Total Growth Rate

Combining the EGF‐dependent growth and density‐dependent inhibition terms, each cell’s net growth rate is:

$$\begin{aligned} {G_{n}}_{total,i}=\delta_{i}\times{G_{n}}_{density,i}\times{G_{n}}_{EGF,i} \#\left( S7 \right) \end{aligned}$$

where $\delta_{i}$​ is the cell’s intrinsic maximal doubling rate (e.g., $\delta_{basal}$or $\delta_{stem}$) given in hours. Finally, cell *i* grows by updating its target volume:

$$\begin{aligned} {{V_{target}}_{n+1}}_{i} ={{V_{target}}_{n}}_{i}+ {G_{n}}_{total,i} \#\left( S8 \right) \end{aligned}$$

## S1.5 Parameter Fitting for Hill Function Coefficients

The half-maximal concentration parameters (${k_{m}}_{EGF}$ and${k_{m}}_{density}$) are critical control points in our growth regulation model, directly influencing proliferation rates in response to both EGF signaling and contact inhibition. To determine appropriate values for these parameters, we employed a multi-objective optimization approach constrained by three key biological criteria:

1. **Epithelial Turnover and Recovery Timeline**: Values were calibrated to ensure that complete epithelium turnover time of 7-14 days, and the recovery of 3-5 days after injury, matched the empirically observed times in healthy corneal epithelium (1,2).
2. **Layer-Specific Cell Density**: Parameter values were adjusted to maintain physiologically relevant cell numbers, 5–7 layers of cells (3).
3. **Epithelial Thickness Maintenance**: We constrained our parameters to maintain stable epithelial thickness of 54 ± 5 μm over extended simulations, consistent with in vivo measurements (3).

We performed an iterative parameter sweep, systematically varying ${k_{m}}_{EGF}$ from 1.0 to 10.0 and ${k_{m}}_{density}$) from 10.0 to 200.0 across multiple simulation runs. The optimal values (${k_{m}}_{EGF, stem}= 3.5$, ${k_{m}}_{EGF, basal}= 7.0$ and ${k_{m}}_{density}$ = 125.0 for both) produced the closest match to all three biological constraints simultaneously.

Throughout this work we fixed the Hill exponent $n=4$for both the EGF-dependent growth term and the density-dependent inhibition term. This choice is motivated by experimental reports that multi-step MAPK/ERK signaling cascades convert graded inputs into ultrasensitive outputs with effective Hill coefficients of approximately 4–5 (4), and by quantitative measurements of contact inhibition in epithelial monolayers, where the dependence of mitotic rate on local cell density is well fit by a Hill function with exponent close to 4 (5). In exploratory simulations we tested smaller exponents ($n=1$–2) and found that they produced graded, “leaky” proliferation, which either prevented the establishment of a stable homeostatic epithelium or led to unrealistic thickening. Fixing $n=4$ provided sufficiently sharp switching to satisfy the three biological constraints described above with a single parameter set.

## S1.6 Mitosis Rule

Mitosis in our model is governed by a volume-threshold mechanism that initiates cell division once a cell accumulates sufficient biomass.

Formally, for any proliferative cell *i* (stem or basal), division occurs when:

$$\begin{aligned} P_{divide}=\left\{ \begin{matrix} 1, & V_{i}> \omega_{v,i} \\ 0, & \mathrm{otherwise} \end{matrix} \right.\#\left( S9 \right) \end{aligned}$$

where $V_{i}$​ represents the current volume of cell, and$\omega_{v,i}$ is the cell type-specific volume threshold for division. We set $\omega_{v,i}=2V_{0_{i}}$where $V_{0_{i}}$​ is the initial cell volume (25 pixels or approximately 100 μm²).

When a cell reaches this threshold, we implement division through the following algorithm:

1. Identify the division plane orientation:

For limbal epithelial stem cells (LESCs), we orient the division plane to direct daughter cells centripetally, following observed patterns that maintain stem cell populations while producing committed progenitors (6).

For basal cells, we select a random division plane to promote even tissue distribution (7).

1. Create two daughter cells with equal volumes:

$$\begin{aligned} V_{d1}=V_{d2}=\frac{V_{p}}{2} \#\left( S10 \right) \end{aligned}$$

where $V_{d1}$​ and $V_{d2}$​ are the volumes of the daughter cells, and $V_{p}$​ is the parent cell's volume at division.

1. Reset target volumes for daughter cells to their initial type-specific values:

$$\begin{aligned} {V_{target}}_{d1}={V_{target}}_{d2}={V_{0}}_{target,i} \#\left( S11 \right) \end{aligned}$$

1. Preserve cell type-specific properties:

Each LESC division produces one stem cell and one basal cell, maintaining the stem cell pool while generating committed progenitors (8).

Basal cell divisions produce two identical basal cells, both capable of further proliferation.

# References

1. Abu-Romman A, Scholand KK, Govindarajan G, Yu Z, Pal-Ghosh S, Stepp MA, et al. Age-Related Differences in the Mouse Corneal Epithelial Transcriptome and Their Impact on Corneal Wound Healing. Invest Ophthalmol Vis Sci. 2024 May 13;65(5):21.

2. Zhu J, Lan X, Mo K, Zhang W, Huang Y, Tan J, et al. Deficiency of SECTM1 impairs corneal wound healing in aging. Aging Cell. 2024 Oct;23(10):e14247.

3. Alghamdi A, Khan MS, Dakhil TA. Understanding Corneal Epithelial Thickness Mapping. Middle East Afr J Ophthalmol. 2023 May 25;29(3):147–55.

4. Huang CY, Ferrell JE. Ultrasensitivity in the mitogen-activated protein kinase cascade. Proceedings of the National Academy of Sciences. 1996 Sept 17;93(19):10078–83.

5. Puliafito A, Hufnagel L, Neveu P, Streichan S, Sigal A, Fygenson DK, et al. Collective and single cell behavior in epithelial contact inhibition. Proceedings of the National Academy of Sciences. 2012 Jan 17;109(3):739–44.

6. Lobo EP, Delic NC, Richardson A, Raviraj V, Halliday GM, Di Girolamo N, et al. Self-organized centripetal movement of corneal epithelium in the absence of external cues. Nat Commun. 2016 Aug 8;7:12388.

7. Beebe DC, Masters BR. Cell lineage and the differentiation of corneal epithelial cells. Invest Ophthalmol Vis Sci. 1996 Aug;37(9):1815–25.

8. De Paiva CS, Pflugfelder SC, Li DQ. Cell Size Correlates with Phenotype and Proliferative Capacity in Human Corneal Epithelial Cells. Stem Cells. 2006 Feb 1;24(2):368–75.
