# Supplementary material for: V-Cornea: A computational model of corneal epithelium homeostasis, injury, and recovery
Source: PLoS Comput Biol. 2025 Dec 26;21(12):e1013410. doi: 10.1371/journal.pcbi.1013410 (PMC12768419; doi:10.1371/journal.pcbi.1013410)
Supplement: S2 Text — Mathematical formulation of the state transitions between cell types, including Stem-to-Basal, Basal-to-Wing, and Wing-to-Superficial differentiation based on neighbor contact and spatial context. (DOCX) [file pcbi.1013410.s002.docx]

S2 Text. V‑Cornea Supplemental Mathematical Formulation for Differentiation Rules.
Manuscript Title: V-Cornea: A computational model of corneal epithelium homeostasis, injury, and recovery
Authors: Joel Vanin ^a^, Michael Getz ^a^, Catherine Mahony ^b^, Thomas B. Knudsen ^a^ & James A. Glazier ^a*^
Affiliations: ^a^ Department of Intelligent Systems Engineering and Biocomplexity Institute, Indiana University, Bloomington, Indiana, United States of America; ^b^ Procter & Gamble Technical Centre, Reading, United Kingdom;

# S2. Differentiation Rules Mathematical Formulation

## S2.1 Stem to Basal Differentiation

A limbal epithelial stem cells (LESCs) differentiates into basal cells it loses contact with limbal Bowman's layer / Epithelial Basement Membrane (EpBM):

$$\begin{aligned} P_{\left( STEM\to BASAL \right)}=\left\{ \begin{matrix} 1, & \mathrm{if} \left( \{LIMB\}\notin N \right) \\ 0, & \mathrm{otherwise} \end{matrix} \right.\#\left( S12 \right) \end{aligned}$$

here $N$ is the set of neighboring cell types, $\neg LIMB \in N$ means the LIMB type is not in the neighbor set.

## S2.2 Basal to Wing Differentiation

Basal cells differentiate to wing cells based on their contact area with Bowman's layer/Epithelial Basement Membrane (EpBM):

$$\begin{aligned} P_{\left( BASAL\to WING \right)}=\left\{ \begin{matrix} 1, & \mathrm{if}A_{EpBM}\leq\omega_{contact,basal} \\ 0, & \mathrm{otherwise} \end{matrix} \right.\#\left( S13 \right) \end{aligned}$$

where $A_{EpBM}$is the number of pixels in contact with the basement membrane, and $\omega_{contact,basal}=5$ pixels are the minimal contact area required to maintain basal phenotype.

## S2.3 Wing to Superficial Differentiation:

A wing cell differentiates into a superficial cell once it contacts the surface (tear film) and loses contact with deeper layers. The transition is:

$$\begin{aligned} P_{\left( \mathrm{WING}\to SUPER \right)}=\left\{ \begin{matrix} 1, & \mathrm{if}\left\{ TEAR,WING \right\}\subset N and \{BASAL, MEMB,STEM\} \notin N \\ 0, & \mathrm{otherwise} \end{matrix} \right.\#\left( S14 \right) \end{aligned}$$

All these transitions are deterministic ($P=1$ or $P=0$) and occur immediately once the condition is met.
