# Supplementary material for: V-Cornea: A computational model of corneal epithelium homeostasis, injury, and recovery
Source: PLoS Comput Biol. 2025 Dec 26;21(12):e1013410. doi: 10.1371/journal.pcbi.1013410 (PMC12768419; doi:10.1371/journal.pcbi.1013410)
Supplement: S3 Text — Definition of the Cellular Potts Model Hamiltonian, including contact energies, Hookean link (spring) constraints, volume/surface constraints, and the chemotaxis algorithm used to control cell motility. (DOCX) [file pcbi.1013410.s003.docx]

S3 Text. V‑Cornea Supplemental Mathematical Formulation for Cellular Movement and Mechanics.
Manuscript Title: V-Cornea: A computational model of corneal epithelium homeostasis, injury, and recovery
Authors: Joel Vanin ^a^, Michael Getz ^a^, Catherine Mahony ^b^, Thomas B. Knudsen ^a^ & James A. Glazier ^a*^
Affiliations: ^a^ Department of Intelligent Systems Engineering and Biocomplexity Institute, Indiana University, Bloomington, Indiana, United States of America; ^b^ Procter & Gamble Technical Centre, Reading, United Kingdom;

# S3. Movement Implementation in the Cellular Potts Model

## S3.1 Hamiltonian Definition

Cell movement is governed by the minimization of the system's Hamiltonian:

$$\begin{aligned} \Delta H={\Delta H}_{contact}{+ \Delta H}_{links}+{\Delta H}_{volume}+{\Delta H}_{surface}+{\Delta H}_{chemotaxis}\#\left( S15 \right) \end{aligned}$$

## S3.2 Contact and Links Energy Terms

The contact energy is calculated as:

$$\begin{aligned} H_{contact}=\sum_{(p,q)} J\left( \tau\left( \sigma\left( p \right) \right),\tau\left( \sigma\left( q \right) \right) \right)\#\left( S16 \right) \end{aligned}$$

where $J(\tau₁,\tau₂)$ is the contact energy between cell types τ₁ and τ₂ and $\sigma(p)$ is the cell occupying lattice site *p*. $\tau(\sigma)$ gives the cell type of cell σ.

Each link is modeled as a Hooke‐type (spring) interaction between the centers of mass of two cells (or between one cell and a “wall” cell). These springs can be created, updated (e.g., to maintain a certain tension), or removed depending on local conditions in the simulation.

$$\begin{aligned} H_{links}={\lambda_{d}}_{i,j}\left( d_{i,j}- {d_{target}}_{i,j} \right)^{2}\#\left( S17 \right) \end{aligned}$$

Where ${\lambda_{d}}_{i,j}$is the link`s spring constant, $d_{i,j}$ is the current distance (in voxels) between the cell centers, ${d_{target}}_{i,j}$is the links equilibrium length.

From this energy, the tension (force) on each cell due to the link is:

$$\begin{aligned} \gamma_{i,j}=\frac{\partial H_{links}}{\partial d_{i,j}}=2\lambda_{i,j}\left( d_{i,j}-d_{target_{i,j}} \right)\#\left( S18 \right) \end{aligned}$$

We enforce a constant tension on the superficial cells layer by automatically adjusting either $\lambda_{i,j}$or $d_{target_{i,j}}$.

$$\begin{aligned} \gamma_{i,j}=2\lambda_{i,j}\left( d_{i,j}-d_{target_{i,j}} \right)=\gamma_{target}\#\left( S19 \right) \end{aligned}$$

**Case of Adjusting** $\boldsymbol{\lambda}_{\boldsymbol{i,j}}$**​**

$$\begin{aligned} \lambda_{i,j}=\frac{\frac{\gamma_{target}}{2}}{d_{i,j}-d_{target_{i,j}}}\#\left( S20 \right) \end{aligned}$$

**Case of Adjusting** $\boldsymbol{d}_{\boldsymbol{targe}\boldsymbol{t}_{\boldsymbol{i,j}}}$**​**

$$\begin{aligned} d_{target_{i,j}}=\frac{\gamma_{target}}{{2\lambda}_{i,j}}\#\left( S21 \right) \end{aligned}$$

Depending on the user’s “AutoAdjustLinks” and “Lambda_link_adjustment” settings, the code picks one of these two strategies to keep $\gamma_{i,j}$ at the desired tension**.**

## S3.3 Volume and Surface Constraints

Volume and surface energy terms are:

$$\begin{aligned} H_{volume}=\lambda_{v}\sum_{i} \left( V_{i}- {V_{target}}_{i} \right)^{2}\#\left( S22 \right) \end{aligned}$$

$$\begin{aligned} H_{surface}=\lambda_{s}\sum_{i} \left( S_{i}- {S_{target}}_{i} \right)^{2}\#\left( S23 \right) \end{aligned}$$

Where $\lambda_{v}, \lambda_{s}$ are volume/surface coefficients;$V_{i}, S_{i}$ are the current volume/surface area. $V_{target_{i}}, S_{target_{i}}$ are the respective targets.

## S3.4 Chemotaxis Term

The chemotactic energy contribution is:

$$\begin{aligned} {\Delta H}_{chemotaxis}=-\lambda_{c}\left( c\left( x^{'} \right)-c\left( x \right) \right)\#\left( S24 \right) \end{aligned}$$

Where $\lambda_{c}$ is the chemotactic coefficient, and $c(x)$ is the chemical concentration at position *x*. A proposed copy from *x* to $x'$changes the Hamiltonian according to $\Delta H$.

## S3.5 Movement Algorithm

1. Randomly choose a lattice pixel *x* and one of its neighbors $x^{'}.$
2. Compute ΔH for copying σ(x)→σ(x′).
3. Accept with probability

$$\begin{aligned} P_{accept}=\left\{ \begin{aligned} 1, & \Delta H \leq0 \\ exp\left( -\frac{\Delta H}{T} \right), \Delta H > 0 \end{aligned} \right.\#\left( S25 \right) \end{aligned}$$

where T is the simulation temperature parameter.
