# Supplementary material for: V-Cornea: A computational model of corneal epithelium homeostasis, injury, and recovery
Source: PLoS Comput Biol. 2025 Dec 26;21(12):e1013410. doi: 10.1371/journal.pcbi.1013410 (PMC12768419; doi:10.1371/journal.pcbi.1013410)
Supplement: S4 Text — Cell Death and Injury Implementation. Mathematical formulation for natural cell turnover (sloughing), volume-based death, and the implementation of specific injury modes including mechanical ablation and chemical burns. (DOCX) [file pcbi.1013410.s004.docx]

S4 Text. V‑Cornea Supplemental Mathematical Formulation for Cell Death and Injury Implementation.
Manuscript Title: V-Cornea: A computational model of corneal epithelium homeostasis, injury, and recovery
Authors: Joel Vanin ^a^, Michael Getz ^a^, Catherine Mahony ^b^, Thomas B. Knudsen ^a^ & James A. Glazier ^a*^
Affiliations: ^a^ Department of Intelligent Systems Engineering and Biocomplexity Institute, Indiana University, Bloomington, Indiana, United States of America; ^b^ Procter & Gamble Technical Centre, Reading, United Kingdom;

# S4. Cell Death and Sloughing Mathematical Formulation

## S4.1 Natural Cell Death (Sloughing)

For superficial cells in contact with tear film, the probability of sloughing is:

$$\begin{aligned} P_{slought}=U\left( 0,1 \right)<\left( \frac{1}{3}*DaytoMCS \right)\#\left( S26 \right) \end{aligned}$$

where $U(0,1)$ is a uniform random sample in [0,1], and $DaytoMCS$ is the factor converting between days and Monte Carlo Steps. The multiplier 1/3 is calibrated so that the average layer transit time is about 1.75 days out of a total 7–14 day turnover cycle.

## S4.2 Volume-Based Death Condition

For superficial cells:

$$\begin{aligned} P_{death}=\left\{ \begin{aligned} 1, &\mathrm{if} {V_{n}}_{i}<\omega_{volume,super} \mathrm{or}N_{super}=0 \\ 0, &\mathrm{otherwise} \end{aligned} \right.\#\left( S27 \right) \end{aligned}$$

where ${V_{n}}_{cell,i}$ is current cell volume, $\omega_{volume,super}= 15$ (minimum viable cell size), $N_{super}$ is the number of neighboring superficial cells.

## S4.3 Volume Reduction During Death

Dying cells shrink to zero volume:

$$\begin{aligned} \frac{\partial V_{i}}{\partial t}=-\lambda_{v_{i}}\left( V_{i}-{V_{target}}_{i} \right)\#\left( S28 \right) \end{aligned}$$

where for a dying cell, $V_{target_{i}}$ = 0 and $\lambda_{v_{i}}$ is increased to 1000 for rapid shrinkage.

## S4.4 Injury Implementation

*A. Ablation Injuries*

Ablation injuries are simulated by the instantaneous removal of cells within a defined circular region. Any part of the cell included in the circular area will be selected to be part of the cells set to be removed, defined as:

$$\begin{aligned} S_{ablate}=\left\{ \left( x,y \right) \right| \sqrt{\left( x-x_{center} \right)^{2}+\left( y-y_{center} \right)^{2}}\leq r \},\#\left( S29 \right) \end{aligned}$$

All cells intersecting $S_{ablate}$ are converted to “tear” type:

$$\begin{aligned} {\forall C \in S}_{ablate},then C_{i}\to C_{tear}\#\left( S30 \right) \end{aligned}$$

simulating the removal of epithelium and its replacement by tear fluid in the wound, as observed in corneal wound healing (1).

*B. Chemical Injuries*

Chemical injuries are modeled through a reaction-diffusion equation:

$$\begin{aligned} \frac{\partial c_{chem}}{\partial t}=\vec{\nabla}\cdot\left( D_{chem}\vec{\nabla}c_{chem} \right)-k_{d_{chem}}*c_{chem}\#\left( S31 \right) \end{aligned}$$

where $c_{chem}(x,y,t)$ is chemical concentration, $D_{chem}$ is the diffusion coefficient that is dependent on localization, $k_{d_{chem}}$ is degradation rate.

With two initial-condition types:

**Gaussian Pulse** (droplet or localized exposure):

$$\begin{aligned} c_{chem}\left( x,y \right)=c_{0_{chem}}*exp\left( -\left( \left( x-x_{center_{chem}} \right)^{2}+\left( y-y_{center_{chem}} \right)^{2} \right)/2\sigma^{2} \right)\#\left( S32 \right) \end{aligned}$$

where $c_{0_{chem}}$ is initial peak concentration, $\sigma$ is distribution width parameter, $(x_{center_{chem}}, y_{center_{chem}})$ is exposure center point.

**Uniform Distribution** (coating or widespread exposure):

$$\begin{aligned} c_{chem}\left( x,y \right)=\frac{c_{0_{chem}}}{L_{x}} for all x\in\left[ 0,L_{x} \right]\times y_{center_{chem}}\#\left( S33 \right) \end{aligned}$$

where $L_{x}$ are the whole lattice dimension in *x*, $y_{center_{chem}}$ is the given height, and $c_{0_{chem}}$ is initial concentration.

## S4.5 Cell Death Conditions

Cells die if their mean chemical concentration exceeds a threshold. Let

$$\begin{aligned} {\vartheta_{n}}_{chem,i}=\sum_{p\in i} F_{chem}\left( p \right)\#\left( S34 \right) \end{aligned}$$

$$\begin{aligned} {{Chem}_{n}}_{i}=\frac{{\vartheta_{n}}_{chem,i}}{{V_{n}}_{i}} \#\left( S35 \right) \end{aligned}$$

$F_{chem}\left( p \right)$ is the chemical field value at pixel $p$ within the cell, ${\vartheta_{n}}_{chem,i}$ is the total amount of chemical observed by the cell $i$ at time $n$, ${V_{n}}_{i}$is the volume of the cell $i$, lastly ${{Chem}_{n}}_{i}$​ is the average concentration of chemical at cell $i$ at time $n$. Which define the set of doomed cells in:

$$\begin{aligned} S_{chem} = \{i | {{Chem}_{n}}_{i} > \omega_{chem}\}\#\left( S36 \right) \end{aligned}$$

All cells in $S_{chem}$, undergo rapid volume collapse:

$$\begin{aligned} \forall C\in S_{chem}:\begin{aligned} \lambda_{v} = 1000 \\ V_{target}=0 \end{aligned}\#\left( S37 \right) \end{aligned}$$

# References

1. Wilson SE, Liang Q, Kim WJ. Lacrimal Gland HGF, KGF, and EGF mRNA Levels Increase after Corneal Epithelial Wounding. Investigative Ophthalmology & Visual Science. 1999 Sept 1;40(10):2185–90.
