# Supplementary material for: V-Cornea: A computational model of corneal epithelium homeostasis, injury, and recovery
Source: PLoS Comput Biol. 2025 Dec 26;21(12):e1013410. doi: 10.1371/journal.pcbi.1013410 (PMC12768419; doi:10.1371/journal.pcbi.1013410)
Supplement: S5 Text — Mathematical description of the spatiotemporal evolution of the Epidermal Growth Factor (EGF) field, including diffusion coefficients, decay rates, boundary conditions, and source/sink terms. (DOCX) [file pcbi.1013410.s005.docx]

S5 Text. V‑Cornea Supplemental Mathematical Formulation for EGF Reaction-Diffusion Dynamics.
Manuscript Title: V-Cornea: A computational model of corneal epithelium homeostasis, injury, and recovery
Authors: Joel Vanin ^a^, Michael Getz ^a^, Catherine Mahony ^b^, Thomas B. Knudsen ^a^ & James A. Glazier ^a*^
Affiliations: ^a^ Department of Intelligent Systems Engineering and Biocomplexity Institute, Indiana University, Bloomington, Indiana, United States of America; ^b^ Procter & Gamble Technical Centre, Reading, United Kingdom;

# S5. EGF Dynamics Mathematical Formulation

## S5.1 Spatiotemporal Evolution

The EGF concentration field $c_{EGF}(x,y,t)$ evolves according to the reaction-diffusion equation:

$$\begin{aligned} \frac{\partial c_{EGF}}{\partial t}=D_{EGF}\left( x,y \right)\nabla^{2}c_{EGF}-k_{d_{EGF}}*c_{EGF}+Ω_{EGF}\left( x,y,t \right)\#\left( S38 \right) \end{aligned}$$

where $D_{EGF}(x,y)$ is the cell-type dependent diffusion coefficient, $k_{d_{EGF}}= 0.5 MCS⁻¹$ $(EGF_{GlobalDecay})$ is the global decay rate, $Ω_{EGF}(x,y,t)$ represents the net source/sink terms.

## S5.2 Cell-Type Dependent Transport

EGF diffusion coefficient varies by cell type:

$$\begin{aligned} D_{EGF}\left( x,y \right)=\left\{ \begin{matrix} 20.0, & in superficial cells\left( D_{EGF,super} \right) \\ 20.0, & in membrane EpBM\left( D_{EGF,memb} \right) \\ 0, & in limbal EpBM\left( D_{EGF,limb} \right) \\ D_{{global}_{EGF}} & \mathrm{otherwise} \end{matrix} \right.\#\left( S39 \right) \end{aligned}$$

$D_{{global}_{EGF}}$ global diffusion constant for EGF was set at 186 voxels²/MCS based on experimental measurements adapted to our simulation parameters. We initially calculated the diffusion coefficient from rat brain tissue studies (1), yielding 466.2 voxels²/MCS after converting to our spatial (1 voxel = 2 μm) and temporal (1 hour = 10 MCS) scales. However, this value would cause unrealistically rapid equilibration across our simulation domain (200 × 90 voxels).

We therefore considered alternative data from GelMA hydrogel experiments (2), which reported a more restricted EGF diffusion coefficient (2.5 × 10⁻⁸ cm²/s), equivalent to 225 voxels²/MCS in our units. Our selected value of 186 voxels²/MCS closely approximates this experimentally determined rate for EGF in dense extracellular environments while remaining computationally feasible. This parameter allows for biologically relevant gradient formation, providing an appropriate balance between physiological accuracy and computational efficiency.

## S5.3 Source and Sink Terms

$$\begin{aligned} Ω_{EGF}\left( x,y,t \right)=\varphi_{EGF,Tear}-k_{d_{EGF}}-\sum_{i} \mu_{EGF,i}*c_{EGF}\left( x,y \right)\#\left( S40 \right) \end{aligned}$$

where $\varphi_{EGF,tear} = 1.0$ is the constant secretion rate by tear cells. And $\mu_{EGF,i}$ are cell-type specific uptake rates $\mu_{EGF,basal}$ = 0.0 for basal cells, $\mu_{EGF,stem}$ = 0.0 for stem cells, $\mu_{EGF,super}$ = 0.0 for superficial cells, $\mu_{EGF,wing}$ = 0.0 for wing cells, since we are using a increase global decay as a surrogate to these individual cells uptakes for simplicity.

The global decay constant for EGF $k_{d_{EGF}}$ was set to 0.5 per MCS to account for multiple biological processes affecting EGF availability. Using published data on EGF circulating half-life ranging from 42 to 114 minutes (3), we calculated corresponding decay constants between 0.099 and 0.036 per MCS in our simulation units (1 hour = 10 MCS).

Our implemented decay value (0.5) is intentionally higher than these physiological baseline values to implicitly incorporate additional processes not explicitly modeled, including cellular uptake, receptor-mediated endocytosis, proteolytic degradation, and sequestration by extracellular matrix components. This higher decay rate also ensures appropriate diffusion length scales within our simulation domain.

## S5.4 Boundary Conditions

**Physical Domain Structure**

The simulation domain is bounded by non-diffusive wall cells at

$$\begin{aligned} x=0 \mathrm{and} x=L_{x} \left( wall cells \right)\#\left( S41 \right) \end{aligned}$$

where wall cells have $D_{EGF,wall} = 0$ (no diffusion). These cells create effective no-flux boundaries by blocking EGF transport.

**Mathematical Boundary Conditions**

*Horizontal Boundaries*

Effective no-flux condition due to combination of:

1. Zero-derivative boundary condition at domain edges:

$$\begin{aligned} \frac{\partial c}{\partial x}=0 \mathrm{at} x=0,L_{x}\#\left( S42 \right) \end{aligned}$$

1. Impermeable wall cells creating physical barriers:

$$\begin{aligned} D=0 in wall cells \mathrm{at} x=0,L_{x}\#\left( S43 \right) \end{aligned}$$

*Vertical Boundaries*

Fixed concentration at top and bottom:

$$\begin{aligned} c=0 \mathrm{at} y=0,L_{y}\#\left( S44 \right) \end{aligned}$$

This configuration creates a physically confined system where:

EGF cannot escape through the lateral boundaries (wall cells + no-flux conditions)

Top and bottom boundaries maintain zero concentration

Effective transport is confined to the region between wall cells

## S5.5 Spatial and Temporal Scales

Spatial:

Lattice spacing: $\Delta x = \Delta y = 1 voxel = 2 \mu m$

Domain size: $L_{x} = 200 voxels, L_{y} = 90 voxels$

Temporal:

1 Monte Carlo Step (MCS) = 6 minutes

# References

1. Thorne RG, Hrabětová S, Nicholson C. Diffusion of Epidermal Growth Factor in Rat Brain Extracellular Space Measured by Integrative Optical Imaging. Journal of Neurophysiology. 2004 Dec;92(6):3471–81.

2. Kuo CY, Eranki A, Placone JK, Rhodes KR, Aranda-Espinoza H, Fernandes R, et al. Development of a 3D Printed, Bioengineered Placenta Model to Evaluate the Role of Trophoblast Migration in Preeclampsia. ACS Biomater Sci Eng. 2016 Oct 10;2(10):1817–26.

3. Chan KY, Lindquist TD, Edenfield MJ, Nicolson MA, Banks AR. Pharmacokinetic study of recombinant human epidermal growth factor in the anterior eye. Invest Ophthalmol Vis Sci. 1991 Dec;32(13):3209–15.
