# Supplementary material for: V-Cornea: A computational model of corneal epithelium homeostasis, injury, and recovery
Source: PLoS Comput Biol. 2025 Dec 26;21(12):e1013410. doi: 10.1371/journal.pcbi.1013410 (PMC12768419; doi:10.1371/journal.pcbi.1013410)
Supplement: S1 Table — Summary of the functional behaviors (growth, mitosis, differentiation, movement) for Stem cells, detailing the mathematical forms, specific signals (EGF, Pressure), and associated model parameters. (DOCX) [file pcbi.1013410.s006.docx]

S1 Table. V‑Cornea supplemental parameters tables
Manuscript Title: V-Cornea: A computational model of corneal epithelium homeostasis, injury, and recovery
Authors: Joel Vanin ^a^, Michael Getz ^a^, Catherine Mahony ^b^, Thomas B. Knudsen ^a^ & James A. Glazier ^a*^
Affiliations: ^a^ Department of Intelligent Systems Engineering and Biocomplexity Institute, Indiana University, Bloomington, Indiana, United States of America; ^b^ Procter & Gamble Technical Centre, Reading, United Kingdom;

*Table S1. Stem cells behavior signal relationship*

| **Agent Type** | **Behavior** | **Form** | $\frac{\boldsymbol{Min}}{\boldsymbol{Max}}$ | **Signal(s)** | **Effect(s)** | **Params** |
| --- | --- | --- | --- | --- | --- | --- |
| **Stem** | Growth  ([Eq. S8](#E8)) | Multiplicative Hill ([Eq. S7](#E7)) | $\frac{0}{\delta_{stem}}$ | EGF ([Eq. S1](#E1)) | Increase | Half max: ${k_{m}}_{EGF,stem}$  Hill power: 4 |
|  |  |  |  | Pressure ([Eq. S4](#E4)) | Decrease | Half max: ${k_{m}}_{density,stem}$  Hill power: 4 |
|  | Differentiation to Basal ([Eq. 12](#E9)) | Boolean Conditional | $0/1$ | Contact with Limbal BM | Disallow | $\omega_{contact,stem}=1$ voxel |
|  | Mitosis | Boolean Conditional | $0/1$ | Cell Volume | Allow | $\omega_{v,stem}=2{V_{0}}_{target, stem}$ |
|  | Movement (Boltzmann Acceptance [Eq. S25](#E22)) | Contact Energy ([Eq. S16](#E13)) | $\frac{5}{20}$ | Cell Neighbor | Energy Contribution | [S6 Table](#TableS6) energies |
|  |  | Volume ([Eq. S22](#E19)) | $\frac{-\infty}{+\infty}$ | Cell Volume | Energy Contribution | $\lambda_{0_{v,stem}}$=2.0,  ${V_{0}}_{target, stem}$=25.0 |
|  |  | Surface Area  ([Eq. S23](#E20)) | $\frac{-\infty}{+\infty}$ | Cell Surface | Energy Contribution | $\lambda_{0_{s,stem}}$=2.0,  ${S_{0}}_{target,stem}$=18.0 |
|  |  | Chemotaxis  ([Eq. S24](#E21)) | $\frac{-\infty}{+\infty}$ | Concentration Gradient | Increase Energy Contribution | $\lambda_{0_{chemo_{Mbias,stem}}}=100$ |
|  | Apoptosis | Boolean Conditional  ([Eq. S36](#E33)) | $0/1$ | Chemical Concentration | Allow | $\omega_{chem}$ |
