# Supplementary material for: V-Cornea: A computational model of corneal epithelium homeostasis, injury, and recovery
Source: PLoS Comput Biol. 2025 Dec 26;21(12):e1013410. doi: 10.1371/journal.pcbi.1013410 (PMC12768419; doi:10.1371/journal.pcbi.1013410)
Supplement: S2 Table — Summary of the functional behaviors for Basal cells, including EGF-dependent growth, contact-mediated differentiation to Wing cells, and mechanical constraints. (DOCX) [file pcbi.1013410.s007.docx]

S2 Table. V‑Cornea supplemental parameters tables
Manuscript Title: V-Cornea: A computational model of corneal epithelium homeostasis, injury, and recovery
Authors: Joel Vanin ^a^, Michael Getz ^a^, Catherine Mahony ^b^, Thomas B. Knudsen ^a^ & James A. Glazier ^a*^
Affiliations: ^a^ Department of Intelligent Systems Engineering and Biocomplexity Institute, Indiana University, Bloomington, Indiana, United States of America; ^b^ Procter & Gamble Technical Centre, Reading, United Kingdom;

*Table S2. Basal cells behavior signal relationship*

| **Agent Type** | **Behavior** | **Form** | $\frac{\boldsymbol{Min}}{\boldsymbol{Max}}$ | **Signal(s)** | **Effect(s)** | **Params** |
| --- | --- | --- | --- | --- | --- | --- |
| **Basal** | Growth  ([Eq. S8](#E8)) | Multiplicative Hill ([Eq. S7](#E7)) | $\frac{0}{\delta_{basal}}$ | EGF ([Eq. S1](#E1)) | Increase | Half max: ${k_{m}}_{EGF,basal}$  Hill power: 4 |
|  |  |  |  | Pressure ([Eq. S4](#E4)) | Decrease | Half max: ${k_{m}}_{density,basal}$  Hill power: 4 |
|  | Differentiation to Wing  ([Eq. S10](#E10)) | Boolean Conditional | $0/1$ | Contact with Periphery BM | Disallow | $\omega_{contact,basal}=5$ voxels |
|  | Mitosis | Boolean Conditional | $0/1$ | Cell Volume | Allow | $\omega_{v,basal}=2{V_{0}}_{target,basal}$ |
|  | Movement (Boltzmann Acceptance [Eq. S25](#E22)) | Contact energy ([Eq. S16](#E13)) | $\frac{5}{20}$ | Cell Neighbor | Energy Contribution | [S6 Table](#TableS6) energies |
|  |  | Volume ([Eq. S22](#E19)) | $\frac{-\infty}{+\infty}$ | Cell Volume | Energy Contribution | $\lambda_{0_{v,basal}}=2.0$, ${V_{0}}_{target,basal}=25.0$ |
|  |  | Surface area  ([Eq. S23](#E20)) | $\frac{-\infty}{+\infty}$ | Cell Surface | Energy Contribution | $\lambda_{0_{s,basal}}=5.0$, ${S_{0}}_{target,basal}=25$.0 |
|  |  | Chemotaxis  ([Eq. S24](#E21)) | $\frac{-\infty}{+\infty}$ | Concentration Gradient | Increase | $\lambda_{0_{chemo_{Mbias,basal}}}=1000$ |
|  | Apoptosis | Boolean Conditional  ([Eq. S36](#E33)) | $0/1$ | Chemical Concentration | Allow | $\omega_{chem}$ |
