# Supplementary material for: V-Cornea: A computational model of corneal epithelium homeostasis, injury, and recovery
Source: PLoS Comput Biol. 2025 Dec 26;21(12):e1013410. doi: 10.1371/journal.pcbi.1013410 (PMC12768419; doi:10.1371/journal.pcbi.1013410)
Supplement: S3 Table — Summary of the functional behaviors for Wing cells, focusing on the differentiation transition to Superficial cells and chemotactic movement. (DOCX) [file pcbi.1013410.s008.docx]

S3 Table. V‑Cornea supplemental parameters tables
Manuscript Title: V-Cornea: A computational model of corneal epithelium homeostasis, injury, and recovery
Authors: Joel Vanin ^a^, Michael Getz ^a^, Catherine Mahony ^b^, Thomas B. Knudsen ^a^ & James A. Glazier ^a*^
Affiliations: ^a^ Department of Intelligent Systems Engineering and Biocomplexity Institute, Indiana University, Bloomington, Indiana, United States of America; ^b^ Procter & Gamble Technical Centre, Reading, United Kingdom;

*Table S3. Wing cells behavior signal relationship*

| **Agent Type** | **Behavior** | **Form** | $\frac{\boldsymbol{Min}}{\boldsymbol{Max}}$ | **Signal(s)** | **Effect(s)** | **Params** |
| --- | --- | --- | --- | --- | --- | --- |
| **Wing** | Differentiation to Superficial ([Eq. S11](#E11)) | Boolean Conditional | $0/1$ | Exposure to Tear, loss contact Basal | Allow | $\omega_{contact,wing-tear}$ >$0$ AND $\omega_{contact,wing-basal}$ < 1 |
|  | Movement (Boltzmann Acceptance [Eq. S25](#E22)) | Contact energy ([Eq. S16](#E13)) | $\frac{5}{15}$ | Cell Neighbor | Energy Contribution | [S6 Table](#TableS6) energies |
|  |  | Volume ([Eq. S22](#E19)) | $\frac{-\infty}{+\infty}$ | Cell Volume | Energy Contribution | $\lambda_{0_{v,wing}}= 2.0$, ${V_{0}}_{target,wing}=25$.0 |
|  |  | Surface area  ([Eq. S23](#E20)) | $\frac{-\infty}{+\infty}$ | Cell Surface | Energy Contribution | $\lambda_{0_{s,wing}}=5.0$, ${S_{0}}_{target,wing}=25.0$ |
|  |  | Chemotaxis  ([Eq. S24](#E21)) | $\frac{-\infty}{+\infty}$ | Protein Concentration Gradient | Increase | $\lambda_{0_{chemo_{EGF,wing}}}=20$ |
|  | Apoptosis | Boolean Conditional  ([Eq. S36](#E33)) | $0/1$ | Chemical Concentration, | Allow | $\omega_{chem}$ |
