# Supplementary material for: V-Cornea: A computational model of corneal epithelium homeostasis, injury, and recovery
Source: PLoS Comput Biol. 2025 Dec 26;21(12):e1013410. doi: 10.1371/journal.pcbi.1013410 (PMC12768419; doi:10.1371/journal.pcbi.1013410)
Supplement: S4 Table — Summary of the functional behaviors for Superficial cells, highlighting unique mechanical constraints (Hookean links between cell centers for tension maintenance) and the probabilistic sloughing mechanism responsible for natural tissue turnover. (DOCX) [file pcbi.1013410.s009.docx]

S4 Table. V‑Cornea supplemental parameters tables
Manuscript Title: V-Cornea: A computational model of corneal epithelium homeostasis, injury, and recovery
Authors: Joel Vanin ^a^, Michael Getz ^a^, Catherine Mahony ^b^, Thomas B. Knudsen ^a^ & James A. Glazier ^a*^
Affiliations: ^a^ Department of Intelligent Systems Engineering and Biocomplexity Institute, Indiana University, Bloomington, Indiana, United States of America; ^b^ Procter & Gamble Technical Centre, Reading, United Kingdom;

*Table S4. Superficial cells behavior signal relationship*

| **Agent Type** | **Behavior** | **Form** | $\frac{\boldsymbol{Min}}{\boldsymbol{Max}}$ | **Signal(s)** | **Effect(s)** | **Params** |
| --- | --- | --- | --- | --- | --- | --- |
| **Superficial** | Movement (Boltzmann Acceptance [Eq. S25](#E22)) | Contact energy ([Eq. S16](#E13)) | $\frac{2}{15}$ | Cell Neighbor | Energy Contribution | [S6 Table](#TableS6) energies |
|  |  | Volume ([Eq. S22](#E19)) | $\frac{-\infty}{+\infty}$ | Cell Volume | Energy Contribution | $\lambda_{0_{v,super}}$=2.0, ${V_{0}}_{target,super}$=25.0 |
|  |  | Surface area  ([Eq. S23](#E20)) | $\frac{-\infty}{+\infty}$ | Cell Surface | Energy Contribution | $\lambda_{0_{s,super}}$=5.0, ${S_{0}}_{target,super}$=25.0 |
|  |  | Links  ([Eq. S17](#E14)) | $\frac{-\infty}{+\infty}$ | Center of Mass Distance | Energy Contribution | $\lambda_{0_{link,super}}=50$  $L_{0_{target_{link,super}}}=3$  $L_{m_{link,super}}=1000$  $L_{{0_{target}}_{link,wall}}=3$  $\lambda_{0_{link,wall}}=50$  $L_{m_{link,wall}}=1000$ |
|  | Apoptosis | Boolean Conditional  ([Eq. S36](#E33)) | 0/1 | Chemical Concentration | Allow | $\omega_{chem}$ |
|  |  | Sloughing  ([Eq. S23](#E23)) | 0/1 | Probability Draw | Allow | $\left( \frac{1}{3}DaytoMCS \right)$ |
