# Supplementary material for: V-Cornea: A computational model of corneal epithelium homeostasis, injury, and recovery
Source: PLoS Comput Biol. 2025 Dec 26;21(12):e1013410. doi: 10.1371/journal.pcbi.1013410 (PMC12768419; doi:10.1371/journal.pcbi.1013410)
Supplement: S5 Table — Summary of the behaviors for non-cellular agents, including EGF secretion by Tear fluid and the vulnerability of Bowman’s membrane/EpBM to chemical exposure. (DOCX) [file pcbi.1013410.s010.docx]

S5 Table. V‑Cornea supplemental parameters tables
Manuscript Title: V-Cornea: A computational model of corneal epithelium homeostasis, injury, and recovery
Authors: Joel Vanin ^a^, Michael Getz ^a^, Catherine Mahony ^b^, Thomas B. Knudsen ^a^ & James A. Glazier ^a*^
Affiliations: ^a^ Department of Intelligent Systems Engineering and Biocomplexity Institute, Indiana University, Bloomington, Indiana, United States of America; ^b^ Procter & Gamble Technical Centre, Reading, United Kingdom;

*Table S5. Tear and Bowman`s/EpBM behavior signal relationship*

| **Agent Type** | **Behavior** | **Form** | $\frac{\boldsymbol{Min}}{\boldsymbol{Max}}$ | **Signal(s)** | **Effect(s)** | **Params** |
| --- | --- | --- | --- | --- | --- | --- |
| **Tear** | Movement (Boltzmann Acceptance [Eq. S25](#E22)) | Contact energy ([Eq. S16](#E13)) | $\frac{0.1}{10}$ | Cell Neighbor | Energy Contribution | [S6 Table](#TableS6) energies |
|  |  | Volume ([Eq. S22](#E19)) | $\frac{-\infty}{+\infty}$ | Cell Volume | Energy Contribution | $\lambda_{0_{v,tear}}$=1.0, ${V_{0}}_{target,tear}$=50.0 |
|  | Secrete EGF | Constant | 1 | $-$ | $-$ | ${\varphi_{EGF}}_{tear}$ |
| **Bowman`s/ EpBM** | Destructible | Boolean Conditional  ([Eq. S36](#E33)) | 0/1 | Chemical Concentration | Allow | $\omega_{chem}$ |
