# Supplementary material for: V-Cornea: A computational model of corneal epithelium homeostasis, injury, and recovery
Source: PLoS Comput Biol. 2025 Dec 26;21(12):e1013410. doi: 10.1371/journal.pcbi.1013410 (PMC12768419; doi:10.1371/journal.pcbi.1013410)
Supplement: S6 Table — Matrix of contact energy values defining the adhesive strength between all pairs of cell types and environmental agents (Medium, Tear, Membranes, Stroma). These parameters govern differential adhesion, driving cell sorting and the maintenance of tissue stratification. (DOCX) [file pcbi.1013410.s011.docx]

S6 Table. V‑Cornea supplemental parameters tables
Manuscript Title: V-Cornea: A computational model of corneal epithelium homeostasis, injury, and recovery
Authors: Joel Vanin ^a^, Michael Getz ^a^, Catherine Mahony ^b^, Thomas B. Knudsen ^a^ & James A. Glazier ^a*^
Affiliations: ^a^ Department of Intelligent Systems Engineering and Biocomplexity Institute, Indiana University, Bloomington, Indiana, United States of America; ^b^ Procter & Gamble Technical Centre, Reading, United Kingdom;

*Table S6 - Contact energy values (arbitrary units)*

| Cell Type | Medium | STEM | LIMB | BASAL | WING | SUPER | MEMB | STROMA | WALL | TEAR |
| --- | --- | --- | --- | --- | --- | --- | --- | --- | --- | --- |
| Medium | 10.0 | 10.0 | 10.0 | 10.0 | 10.0 | 10.0 | 10.0 | 10.0 | 5.0 | 5.0 |
| STEM |  | 10.0 | 5.0 | 10.0 | 10.0 | 10.0 | 5.0 | 20.0 | 10.0 | 5.0 |
| LIMB |  |  | 10.0 | 10.0 | 10.0 | 10.0 | 10.0 | 10.0 | 10.0 | 5.0 |
| BASAL |  |  |  | 10.0 | 10.0 | 15.0 | 5.0 | 20.0 | 10.0 | 5.0 |
| WING |  |  |  |  | 5.0 | 6.0 | 15.0 | 10.0 | 10.0 | 5.0 |
| SUPER |  |  |  |  |  | 2.0 | 15.0 | 10.0 | 5.0 | 2.0 |
| MEMB |  |  |  |  |  |  | 10.0 | 10.0 | 10.0 | 2.0 |
| STROMA |  |  |  |  |  |  |  | 10.0 | 10.0 | 10.0 |
| WALL |  |  |  |  |  |  |  |  | 10.0 | 5.0 |
| TEAR |  |  |  |  |  |  |  |  |  | 0.1 |
