# Supplementary material for: V-Cornea: A computational model of corneal epithelium homeostasis, injury, and recovery
Source: PLoS Comput Biol. 2025 Dec 26;21(12):e1013410. doi: 10.1371/journal.pcbi.1013410 (PMC12768419; doi:10.1371/journal.pcbi.1013410)
Supplement: S7 Table — Description of the diffusive fields used in the model (EGF, Movement Bias, and Chemical Injury), including their biological roles, units, and transport processes. (DOCX) [file pcbi.1013410.s012.docx]

S7 Table. V‑Cornea supplemental parameters tables
Manuscript Title: V-Cornea: A computational model of corneal epithelium homeostasis, injury, and recovery
Authors: Joel Vanin ^a^, Michael Getz ^a^, Catherine Mahony ^b^, Thomas B. Knudsen ^a^ & James A. Glazier ^a*^
Affiliations: ^a^ Department of Intelligent Systems Engineering and Biocomplexity Institute, Indiana University, Bloomington, Indiana, United States of America; ^b^ Procter & Gamble Technical Centre, Reading, United Kingdom;

*Table S7 – Fields descriptions*

| Field | Definition | Role / Properties | Units | Processes |
| --- | --- | --- | --- | --- |
| $\boldsymbol{F}_{\boldsymbol{EGF}}$ | long-diffusing proliferative factor | -regulates cell growth | nM | -diffuses  -decays |
| $\boldsymbol{F}_{\boldsymbol{Mbias}}$ | short-diffusing chemoattractant | -regulates cell movement | nM | -diffuses  -uptake by Basal and Stem |
| $\boldsymbol{F}_{\boldsymbol{chem}}$ | long-diffusing chemical injury | -regulates cell viability | scaled concentration (amount per voxel) | -diffuses  -uptake (cleared) by Tear  -uptake (cleared) by cell death |
