# Supplementary material for: V-Cornea: A computational model of corneal epithelium homeostasis, injury, and recovery
Source: PLoS Comput Biol. 2025 Dec 26;21(12):e1013410. doi: 10.1371/journal.pcbi.1013410 (PMC12768419; doi:10.1371/journal.pcbi.1013410)
Supplement: S8 Table — Listing of the initial parameter values governing target volumes, surface areas, and growth regulation (Hill function coefficients) for all cell types (Stem, Basal, Wing, Superficial), with literature references. (DOCX) [file pcbi.1013410.s013.docx]

S8 Table. V‑Cornea supplemental parameters tables
Manuscript Title: V-Cornea: A computational model of corneal epithelium homeostasis, injury, and recovery
Authors: Joel Vanin ^a^, Michael Getz ^a^, Catherine Mahony ^b^, Thomas B. Knudsen ^a^ & James A. Glazier ^a*^
Affiliations: ^a^ Department of Intelligent Systems Engineering and Biocomplexity Institute, Indiana University, Bloomington, Indiana, United States of America; ^b^ Procter & Gamble Technical Centre, Reading, United Kingdom;

*Table S8 - Initial parameters (Cell Growth and Mechanical Constraints)*

| **Parameter** | **Symbol** | **Simulation Value** | **Literature**  **Ref.** | **Description** |
| --- | --- | --- | --- | --- |
| **Stem Cells** |  |  |  |  |
| InitSTEM_LambdaSurface | ${\lambda_{0}}_{s,stem}$ | *2.0* | $-$ | *How strongly stem cell regulates its surface area towards a desired size* |
| InitSTEM_TargetSurface | ${S_{0}}_{target,stem}$ | *18.0* | (1) | *The ideal surface area each stem cell tries to maintain. Defined to constraint cell shape* |
| InitSTEM_LambdaVolume | ${\lambda_{0}}_{v,stem}$ | *2.0* | $-$ | *How strongly a stem cell regulates its volume towards a desired size.* |
| InitSTEM_TargetVolume | ${V_{0}}_{target,stem}$ | *25.0* | (2) | *The ideal volume each stem cell tries to maintain. Reference 10 -30 µm* |
| DensitySTEM_HalfMaxValue | ${k_{m}}_{density,stem}$ | *125.0* | *Fitted* [*(S1 Text section S1.5)*](#_S1.5_Parameter_Fitting) | *Cell density of stem cells half-max growth response* |
| EGF_STEM_HalfMaxValue | ${k_{m}}_{EGF,stem}$ | *3.5* | *Fitted* [*(S1 Text section S1.5)*](#_S1.5_Parameter_Fitting) | *EGF concentration stem cells achieve half-max growth response* |
| InitSTEM_LambdaChemo | ${\lambda_{chemo}}_{Mbias,stem}$ | *100.0* | $-$ | *How strongly stem cells move toward areas of higher chemoattract.* |
| **Basal Cells** |  |  |  |  |
| InitBASAL_LambdaSurface | ${\lambda_{0}}_{s,basal}$ | *2.0* | $-$ | *How strongly basal cell regulate its surface area towards a desired size* |
| InitBASAL_TargetSurface | ${S_{0}}_{target,basal}$ | *20.0* | (1) | *The ideal surface area each basal cell tries to maintain. Defined to constraint cell shape* |
| InitBASAL_LambdaVolume | ${\lambda_{0}}_{v,basal}$ | *2.0* | $-$ | *How strongly a basal cell regulates its volume towards a desired size* |
| InitBASAL_TargetVolume | ${V_{0}}_{target,basal}$ | *25.0* | (2) | *The ideal volume each basal cell tries to maintain. Reference 10 -30 µm* |
| DensityBASAL_HalfMaxValue | ${k_{m}}_{density,basal}$ | *125.0* | *Fitted* [*(S1 Text section S1.5)*](#_S1.5_Parameter_Fitting) | *Cell density of basal cells half-max growth response* |
| EGF_BASAL_HalfMaxValue | ${k_{m}}_{EGF,basal}$ | *7.0* | *Fitted* [*(S1 Text section S1.5)*](#_S1.5_Parameter_Fitting) | *EGF concentration basal cells achieve half-max growth response* |
| InitBASAL_LambdaChemo | ${\lambda_{chemo}}_{Mbias,basal}$ | *1000.0* | $-$ | *How strongly basal cells move to areas with more chemoattractant* |
| **Wing Cells** |  |  |  |  |
| InitWING_LambdaSurface | ${\lambda_{0}}_{s,wing}$ | *5.0* | $-$ | *How strongly wing cell regulates its surface area towards a desired size* |
| InitWING_TargetSurface | ${S_{0}}_{target,wing}$ | *25.0* | (1) | *The ideal surface area each wing cell tries to maintain. Defined to constraint cell shape* |
| InitWING_LambdaVolume | ${\lambda_{0}}_{v,wing}$ | *2.0* | $-$ | *How strongly a wing cell regulates its volume towards a desired size* |
| InitWING_TargetVolume | ${V_{0}}_{target,wing}$ | *25.0* | (2) | *The ideal volume each wing cell tries to maintain. Reference 10 -30 µm* |
| InitWING_EGFLambdaChemo | ${\lambda_{chemo}}_{EGF,wing}$ | *20.0* | (3) | *How strongly wing cells move toward regions with higher EGF* |
| **Superficial Cells** |  |  |  |  |
| InitSUPER_LambdaSurface | ${\lambda_{0}}_{s,super}$ | *5.0* | $-$ | *How strongly superficial cells regulate surface area to desired size* |
| InitSUPER_TargetSurface | ${S_{0}}_{target,super}$ | *25.0* | (1) | *The ideal surface area each superficial cell tries to maintain. Defined to constraint cell shape* |
| InitSUPER_LambdaVolume | ${\lambda_{0}}_{v,super}$ | *5.0* | $-$ | *How strongly superficial cells regulate its volume to desired size* |
| InitSUPER_TargetVolume | ${V_{0}}_{target,super}$ | *25.0* | (2) | *Ideal volume superficial cell tries to maintain. Reference 10 -30 µm* |

# References

1. Sterenczak KA, Winter K, Sperlich K, Stahnke T, Linke S, Farrokhi S, et al. Morphological characterization of the human corneal epithelium by in vivo confocal laser scanning microscopy. Quant Imaging Med Surg. 2021 May;11(5):1737–50.

2. De Paiva CS, Pflugfelder SC, Li DQ. Cell Size Correlates with Phenotype and Proliferative Capacity in Human Corneal Epithelial Cells. Stem Cells. 2006 Feb 1;24(2):368–75.

3. Boisjoly HM, Laplante C, Bernatchez SF, Salesse C, Giasson M, Joly MC. Effects of EGF, IL-1 and their Combination on In Vitro Corneal Epithelial Wound Closure and Cell Chemotaxis. Experimental Eye Research. 1993 Sept 1;57(3):293–300.
