# Supplementary material for: V-Cornea: A computational model of corneal epithelium homeostasis, injury, and recovery
Source: PLoS Comput Biol. 2025 Dec 26;21(12):e1013410. doi: 10.1371/journal.pcbi.1013410 (PMC12768419; doi:10.1371/journal.pcbi.1013410)
Supplement: S9 Table — Parameter values for the diffusion coefficients of EGF, Chemical Injury (SLS), and Movement Bias across different cell types and tissue layers, including uptake and secretion rates. (DOCX) [file pcbi.1013410.s014.docx]

S9 Table. V‑Cornea supplemental parameters tables
Manuscript Title: V-Cornea: A computational model of corneal epithelium homeostasis, injury, and recovery
Authors: Joel Vanin ^a^, Michael Getz ^a^, Catherine Mahony ^b^, Thomas B. Knudsen ^a^ & James A. Glazier ^a*^
Affiliations: ^a^ Department of Intelligent Systems Engineering and Biocomplexity Institute, Indiana University, Bloomington, Indiana, United States of America; ^b^ Procter & Gamble Technical Centre, Reading, United Kingdom;

*Table S9 - Initial parameters (Chemical Fields [EGF, SLS, Movement Bias])*

| **Parameter** | **Symbol** | **Simulation Value** | **Literature**  **Ref.** | **Description** |
| --- | --- | --- | --- | --- |
| **SLS Diffusion** |  |  |  |  |
| SLS_STEMDiffCoef | ${D_{chem}}_{stem}$ | *5.0* | $-$ | *How quickly the chemical spreads inside stem cells* |
| SLS_BASALDiffCoef | ${D_{chem}}_{basal}$ | *5.0* | $-$ | *How quickly the chemical spreads inside basal cells* |
| SLS_WINGDiffCoef | ${D_{chem}}_{wing}$ | *5.0* | $-$ | *How quickly the chemical spreads inside wing cells* |
| SLS_SUPERDiffCoef | ${D_{chem}}_{super}$ | *5.0* | $-$ | *How quickly the chemical spreads inside superficial cells* |
| SLS_MEMBDiffCoef | ${D_{chem}}_{memb}$ | *5.0* | $-$ | *How quickly the chemical spreads in the Bowman's membrane (periphery)* |
| SLS_LIMBDiffCoef | ${D_{chem}}_{limb}$ | *5.0* | $-$ | *How quickly chemical spreads in the Bowman's membrane (limbal region)* |
| SLS_TEARDiffCoef | ${D_{chem}}_{tear}$ | *5.0* | $-$ | *How quickly the chemical spreads in the tear layer* |
| SLS Global Decay | ${k_{d}}_{chem}$ | *0.0* | $-$ | *For the model the only breakdown and inactivation of the chemical occurs by cell death where the amount inside the cell gets inactive as cells die and washed by the tear* |
| **EGF Diffusion / Uptake** |  |  |  |  |
| EGF_SUPERDiffCoef | ${D_{EGF}}_{super}$ | *20.0* | (1,2) | *How quickly EGF diffuses (spreads) through superficial cells. The references show a limitation of space for EGF to defuse through the sides of the cells and it was shown that EGF applied in intact corneas do not proliferate as wounded ones* |
| EGF_FieldUptakeBASAL | ${\mu_{EGF}}_{basal}$ | *0.0* | $-$ | *How quickly basal cells consume EGF from their surroundings* |
| EGF_FieldUptakeSTEM | ${\mu_{EGF}}_{stem}$ | *0.0* | $-$ | *How quickly stem cells consume EGF from their surroundings* |
| EGF_FieldUptakeSuper | ${\mu_{EGF}}_{super}$ | *0.0* | $-$ | *How quickly superficial cells consume EGF* |
| EGF_FieldUptakeWing | ${\mu_{EGF}}_{wing}$ | *0.0* | $-$ | *How quickly wing cells consume EGF* |
| EGF_ScreteAmount | ${\varphi_{EGF}}_{tear}$ | *1.0* | (3,4) | *The constant amount of EGF introduced into the system from tears. The value for secretion was normalized to have the max value of 1 throughout the simulated space.* |
| EGF_GlobalDecay | ${k_{d}}_{EGF}$ | *0.5* | (5) | *The overall rate at which EGF naturally breaks down (decays) over time. The values of EGF half-life from the reference was 78 min +- 36 min transformed into* *≈ 0.1 per MCS, value used is higher to account for other things like uptake, evaporation and sequestration.* |
| **Movement Bias Field** |  |  |  |  |
| MovementBiasScreteAmount | ${\varphi_{Mbias}}_{memb, limb}$ | *1.0* | $-$ | *The constant amount of chemoattractant secreted by the boundary (Bowman's membrane) to guide cell movement.* |
| MovementBiasUptake | ${\mu_{Mbias}}_{basal,stem}$ | *1.0* | $-$ | *How quickly basal cells absorb (take up) this chemoattractant* |

# References

1. Savage CR, Cohen S. Proliferation of corneal epithelium induced by epidermal growth factor. Experimental Eye Research. 1973 Mar 1;15(3):361–6.

2. Van Itallie CM, Holmes J, Bridges A, Gookin JL, Coccaro MR, Proctor W, et al. The density of small tight junction pores varies among cell types and is increased by expression of claudin-2. Journal of Cell Science. 2008 Feb 1;121(3):298–305.

3. Peterson JL, Ceresa BP. Epidermal Growth Factor Receptor Expression in the Corneal Epithelium. Cells. 2021 Sept 13;10(9):2409.

4. Rao K, Farley WJ, Pflugfelder SC. Association between High Tear Epidermal Growth Factor Levels and Corneal Subepithelial Fibrosis in Dry Eye Conditions. Investigative Ophthalmology & Visual Science. 2010 Feb 1;51(2):844–9.

5. Chan KY, Lindquist TD, Edenfield MJ, Nicolson MA, Banks AR. Pharmacokinetic study of recombinant human epidermal growth factor in the anterior eye. Invest Ophthalmol Vis Sci. 1991 Dec;32(13):3209–15.
