# Supplementary material for: V-Cornea: A computational model of corneal epithelium homeostasis, injury, and recovery
Source: PLoS Comput Biol. 2025 Dec 26;21(12):e1013410. doi: 10.1371/journal.pcbi.1013410 (PMC12768419; doi:10.1371/journal.pcbi.1013410)
Supplement: S10 Table — Settings for simulating injuries (ablation coordinates, chemical concentration/timing) and the Focal Point Plasticity (FPP) parameters used to model tension at the tissue boundaries. (DOCX) [file pcbi.1013410.s015.docx]

S10 Table. V‑Cornea supplemental parameters tables
Manuscript Title: V-Cornea: A computational model of corneal epithelium homeostasis, injury, and recovery
Authors: Joel Vanin ^a^, Michael Getz ^a^, Catherine Mahony ^b^, Thomas B. Knudsen ^a^ & James A. Glazier ^a*^
Affiliations: ^a^ Department of Intelligent Systems Engineering and Biocomplexity Institute, Indiana University, Bloomington, Indiana, United States of America; ^b^ Procter & Gamble Technical Centre, Reading, United Kingdom;

*Table S10 - Initial parameters (Injury Setup and Focal Point Plasticity Links)*

| **Parameter** | **Symbol** | **Simulation Value** | **Literature**  **Ref.** | **Description** |
| --- | --- | --- | --- | --- |
| SimTime | $t_{final}$ | *50,400.0* | $-$ | *How long the simulation runs before it stops (in MCS)* |
| **Injury Setup** |  |  |  |  |
| InjuryTime | $t_{injury}$ | *7200.0* | $-$ | *The simulation time at which the injury event occurs* |
| SLS_X_Center | ${x_{center}}_{chem}$ | *100* | $-$ | *The x-coordinate of the center of the chemical source* |
| SLS_Y_Center | ${y_{center}}_{chem}$ | *75* | $-$ | *The y-coordinate of the center of the chemical source* |
| SLS_Concentration | $c_{chem}$ | **[750 a.u.(slight) , 1500 a.u.(mild), 2500 a.u.(mod.)]* | (1) | *The initial strength or concentration of the chemical introduced. This parameter was chosen based on the descriptions of different classifications following the depth of injury model* |
| InjuryX_Center | ${x_{center}}_{abla}$ | *150* | $-$ | *The x-coordinate of the ablation injury center* |
| InjuryY_Center | ${y_{center}}_{abla}$ | *60* | $-$ | *The y-coordinate of the ablation injury center* |
| InjuryRadius | $\pi_{abla}$ | *25* | $-$ | *How far from the center cells are affected by the ablation (a radius)* |
| **FPP Link Setup** |  |  |  |  |
| LINKWALL_lambda_distance | $\lambda_{0_{link,wall}}$ | *50* | $-$ | *How strongly the link between superficial cells and simulation boundary maintains a set distance* |
| LINKWALL_target_distance | $L_{{0_{target}}_{link,wall}}$ | *3* | $-$ | *Desired length of the link between superficial cells and the boundary* |
| LINKWALL_max_distance | $L_{m_{link,wall}}$ | *1000* | $-$ | *Max length link between superficial cells vs boundary can stretch before it breaks* |
| LINKSUPER_lambda_distance | $\lambda_{0_{link,super}}$ | *50* | $-$ | *How strongly the link between two superficial cells maintains a set distance* |
| LINKSUPER_target_distance | $L_{0_{{target}_{link,super}}}$ | *3* | $-$ | *The desired resting length of the link between superficial cells* |
| LINKSUPER_max_distance | $L_{m_{link,super}}$ | *1000* | $-$ | *Max length the link between superficial cells can stretch before it breaks* |

**a.u. is short for arbitrary units in this context*

# References

1. Scott L, Eskes C, Hoffmann S, Adriaens E, Alepée N, Bufo M, et al. A proposed eye irritation testing strategy to reduce and replace *in vivo* studies using Bottom–Up and Top–Down approaches. Toxicology in Vitro. 2010 Feb 1;24(1):1–9.
