# Supplementary material for: V-Cornea: A computational model of corneal epithelium homeostasis, injury, and recovery
Source: PLoS Comput Biol. 2025 Dec 26;21(12):e1013410. doi: 10.1371/journal.pcbi.1013410 (PMC12768419; doi:10.1371/journal.pcbi.1013410)
Supplement: S11 Table — List of boolean flags and settings used to enable/disable specific biological modules (mitosis, death) and configure output metrics (thickness plotting, cell counts) for debugging and analysis. (DOCX) [file pcbi.1013410.s016.docx]

S11 Table. V‑Cornea supplemental parameters tables
Manuscript Title: V-Cornea: A computational model of corneal epithelium homeostasis, injury, and recovery
Authors: Joel Vanin ^a^, Michael Getz ^a^, Catherine Mahony ^b^, Thomas B. Knudsen ^a^ & James A. Glazier ^a*^
Affiliations: ^a^ Department of Intelligent Systems Engineering and Biocomplexity Institute, Indiana University, Bloomington, Indiana, United States of America; ^b^ Procter & Gamble Technical Centre, Reading, United Kingdom;

*Table S11 - Initial parameters (Simulation/Logging Controls)*

| **Parameter** | **Symbol** | **Simulation Value** | **Description** |
| --- | --- | --- | --- |
| GrowthControl | $-$ | *True* | *Enable or disable cell growth processes for debugging* |
| MitosisControl | $-$ | *True* | *Enable or disable cell division (mitosis) for debugging* |
| DeathControl | $-$ | *True* | *Enable or disable cell death mechanisms for debugging* |
| DifferentiationControl | $-$ | *True* | *Enable or disable cell differentiation processes for debugging* |
| CellCount | $-$ | *True* | *Collect the number of cells over time* |
| PressureTracker | $-$ | *False* | *Show a real-time plot of pressure in the simulation* |
| EGF_SeenByCell | $-$ | *True* | *Track the EGF concentration experienced by each cell inside CC3D* |
| SLS_SeenByCell | $-$ | *False* | *Track the chemical concentration experienced by each cell inside CC3D* |
| ThicknessPlot | $-$ | *True* | *Collect and plot data on the tissue thickness over time* |
| SurfactantTracking | $-$ | *False* | *Track chemical distribution over time* |
| SnapShot | $-$ | *True* | *Take snapshots of the simulation state at regular intervals* |
| InjuryType | $-$ | *True* | *Choose the type of injury (e.g., ablation or chemical)* |
| IsInjury | $-$ | *False* | *Enable or disable the injury feature in the simulation* |
| SLS_Threshold | $\omega_{chem}$ | *True* | *The chemical concentration limit above which cells are considered dead* |
| SLS_Injury | $-$ | *False* | *Enable or disable a chemical-based injury using SLS (for testing how chemicals spread and affect cells)* |
| SLS_Threshold_Method | $-$ | *True* | *If enabled, cells will die when chemical level surpasses a certain threshold* |
| SLS_Gaussian_pulse | $-$ | *True* | *Whether the chemical is introduced as a concentrated 'droplet' (Gaussian) or as a uniform 'coating'* |
| AutoAdjustLinks | $-$ | *True* | *If enabled, the link properties are auto adjusted to keep the tension constant* |
