# Supplementary material for: V-Cornea: A computational model of corneal epithelium homeostasis, injury, and recovery
Source: PLoS Comput Biol. 2025 Dec 26;21(12):e1013410. doi: 10.1371/journal.pcbi.1013410 (PMC12768419; doi:10.1371/journal.pcbi.1013410)
Supplement: S12 Table — A detailed comparison of V-Cornea model predictions versus experimental/clinical literature values for key physiological features (epithelial thickness, turnover time, healing rates) serving as the primary validation metrics. (DOCX) [file pcbi.1013410.s017.docx]

S12 Table. V‑Cornea supplemental parameters tables
Manuscript Title: V-Cornea: A computational model of corneal epithelium homeostasis, injury, and recovery
Authors: Joel Vanin ^a^, Michael Getz ^a^, Catherine Mahony ^b^, Thomas B. Knudsen ^a^ & James A. Glazier ^a*^
Affiliations: ^a^ Department of Intelligent Systems Engineering and Biocomplexity Institute, Indiana University, Bloomington, Indiana, United States of America; ^b^ Procter & Gamble Technical Centre, Reading, United Kingdom;

*Table S12 - Expanded version of Table 2, providing detailed descriptions of key corneal features, their experimental/clinical values, V-Cornea predictions, and how each feature was used in model calibration and validation.*

| Feature | Experimental / clinical value (range, with refs) | V-Cornea prediction (this study) | How used in model |
| --- | --- | --- | --- |
| Epithelial thickness and limbal–central gradient | Human corneal epithelium ≈48–55 μm thick, 5–7 cell layers in the central cornea. (1–3) limbal epithelium ~83 μm μm (4–6). | The model produces a stable thickness of 50–54 μm with 5–7 layers at homeostasis, and a thicker epithelium (up to 7–8 layers) over the limbal stem cell region, reproducing a limbal–central thickness difference (Figs. 3 and 8). | **Emergent validation.** Cell sizes and mechanical parameters were set within physiologically plausible ranges based on in vitro measurements and corneal imaging (S8–S11 Tables), but neither absolute epithelial thickness nor a limbal–central difference was directly imposed. Both features emerge from the interaction of growth, differentiation, and mechanical rules acting on these physiologically bounded cells. |
| Epithelial turnover time | Estimates based on in vivo labeling and imaging studies suggest full corneal epithelial turnover in roughly **7–10 days**, with some reports up to **14 days**; direct quantitative data are limited, but multiple kinetic studies are consistent with this range (2,7–9). | The model shows ~7–14 days for complete renewal of superficial layers in the central and peripheral epithelium. | **Constrained calibration.** We used the 7–14 day turnover window as a primary calibration target when tuning proliferation rates and superficial sloughing rate (S8–S11 Tables). Only parameter sets that produced full renewal within this window and preserved realistic epithelial thickness and limbal–central gradients (Row 1) were accepted, ensuring that fitted parameters remained within physiologically reasonable ranges***.*** |
| Healing time, slight/mild epithelial injury (intact BM) | Small/superficial corneal abrasions usually heal in 1–3 days; and larger but still epithelial-only abrasions heal within about 3–5 days (10–13). Here ‘healing’ refers to structural re-epithelialization and restoration of a continuous epithelial surface, as assessed by disappearance of fluorescein-stained defects and normalization of epithelial thickness in clinical imaging. | Slight and mild injuries confined to the epithelium close completely in ~3–5 days, with re-establishment of a continuous epithelial surface and return of thickness and cell counts to pre-injury baseline (Fig. 9–10, top/middle rows). | **Secondary constraint + validation.** During parameter screening, we rejected parameter sets for which slight/mild epithelial-only injuries failed to close within the 1–5 day clinical window (Row 3, experimental column). However, we did not fit an exact time course. After fixing parameters based on homeostasis and turnover (Rows 1–2), we confirmed that the resulting wound-closure dynamics, recovery of epithelial thickness and cell counts, and near-exponential decrease in defect area qualitatively match clinical observations of epithelial sheet migration and area closure. |
| Healing after basement membrane damage (moderate injuries) | When the epithelial basement membrane is disrupted, re-epithelialization and restoration of stable epithelial adhesion are slower and prone to recurrent erosions; clinically, structurally closed epithelia may repeatedly break down for weeks to months, resulting in recurrent corneal erosion (RCE), where structurally closed epithelia reopen under minor stress (11–15) | Moderate injuries that destroy EpBM do not fully return to stable homeostasis over 20 days. After initial closure, the model exhibits repeated episodes of epithelial detachment and loss of superficial/wing layers, with incomplete recovery of cell numbers and thickness—behavior qualitatively similar to clinical RCE. | **Qualitative emergent behavior.** Recurrent breakdown of the epithelium arises naturally from impaired basement membrane adhesion. For example, we did not add any extra rules (e.g., special “RCE” states or injury-depth-dependent sloughing); the same parameter set calibrated for homeostasis and epithelial-only injuries (Rows 1–3) automatically produces RCE-like instability when EpBM is removed. |

# References

1. AlTurki HS, Alsubhi SS, Alhazmi A, Alhadlag A, Albalawi NS, Alzoman MA, et al. Corneal Epithelial Thickness Mapping in Healthy Population Corneas Using MS-39 Anterior Segment Optical Coherence Tomography. OPTH. 2025 Jan 22;19:249–59.

2. Sridhar MS. Anatomy of cornea and ocular surface. Indian J Ophthalmol. 2018 Feb;66(2):190–4.

3. Liang Q, Le Q, Cordova DW, Tseng CH, Deng SX. Corneal Epithelial Thickness Measured Using AS-OCT as a Diagnostic Parameter for Limbal Stem Cell Deficiency. Am J Ophthalmol. 2020 Aug;216:132–9.

4. Feng Y, Simpson TL. Comparison of Human Central Cornea and Limbus in Vivo Using Optical Coherence Tomography. Optometry and Vision Science. 2005 May;82(5):416.

5. Reinstein DZ, Archer TJ, Gobbe M, Silverman RH, Coleman DJ. Epithelial Thickness in the Normal Cornea: Three-dimensional Display With Very High Frequency Ultrasound. J Refract Surg. 2008 June;24(6):571–81.

6. Francoz M, Karamoko I, Baudouin C, Labbé A. Ocular Surface Epithelial Thickness Evaluation with Spectral-Domain Optical Coherence Tomography. Invest Ophthalmol Vis Sci. 2011 Nov 24;52(12):9116–23.

7. Douvaras P, Mort RL, Edwards D, Ramaesh K, Dhillon B, Morley SD, et al. Increased Corneal Epithelial Turnover Contributes to Abnormal Homeostasis in the Pax6+/− Mouse Model of Aniridia. PLOS ONE. 2013 Aug 13;8(8):e71117.

8. Tang G, Chi M, Zhai Y, Peng R, Hong J. Corneal Epithelial Tissue Engineering Strategy Based on Cell Viability Optimization: A Review and Prospects. Bioengineering. 2025 Nov;12(11):1175.

9. Cenedella RJ, Fleschner CR. Kinetics of corneal epithelium turnover in vivo. Studies of lovastatin. Invest Ophthalmol Vis Sci. 1990 Oct;31(10):1957–62.

10. Dua HS, Forrester JV. Clinical patterns of corneal epithelial wound healing. Am J Ophthalmol. 1987 Nov 15;104(5):481–9.

11. Le Sage N, Verreault R, Rochette L. Efficacy of eye patching for traumatic corneal abrasions: a controlled clinical trial. Ann Emerg Med. 2001 Aug;38(2):129–34.

12. Roggeband R, York M, Pericoi M, Braun W. Eye irritation responses in rabbit and man after single applications of equal volumes of undiluted model liquid detergent products. Food Chem Toxicol. 2000 Aug;38(8):727–34.

13. Allen NE, Crawford AZ, McGhee CNJ, Meyer JJ. Chemical eye injuries: a 10 year retrospective review of acute presentations and clinical outcomes in Auckland, New Zealand. Scientific Reports. 2024 Apr 9;14(1):8264.

14. Ramamurthi S, Rahman MQ, Dutton GN, Ramaesh K. Pathogenesis, clinical features and management of recurrent corneal erosions. Eye. 2006 June;20(6):635–44.

15. Eke T, Morrison DA, Austin DJ. Recurrent symptoms following traumatic corneal abrasion: prevalence, severity, and the effect of a simple regimen of prophylaxis. Eye (Lond). 1999 June;13 ( Pt 3a):345–7.
