# Supplementary material for: V-Cornea: A computational model of corneal epithelium homeostasis, injury, and recovery
Source: PLoS Comput Biol. 2025 Dec 26;21(12):e1013410. doi: 10.1371/journal.pcbi.1013410 (PMC12768419; doi:10.1371/journal.pcbi.1013410)
Supplement: Attachment — Submitted filename: Response to the reviewers .docx [file pcbi.1013410.s020.docx]

Response to the reviewers

Reviewers' comments:

Reviewer's Responses to Questions

Reviewer #1: In this work the authors present V-Cornea, an agent-based computational model built in CompuCell3D to simulate corneal epithelial homeostasis and injury response, addressing limitations of current ocular irritation assessments, particularly in predicting long-term effects and recovery. The model integrates biologically inspired rules for cell behaviors and key signaling pathways such as EGF, enabling in vitro to in vivo extrapolation of tissue-level outcomes.

Overall, V-Cornea demonstrates potential as a virtual-tissue platform for toxicological testing, drug discovery, and therapeutic optimization across diverse corneal injury scenarios.

The paper would benefit from three minor revisions:

**1) Add more comparisons between the model prediction and experimental data in a clear figure that demonstrates that the model recapitulates experimental data with physiological model parameters. Adding such a figure is crucial.**

<https://drive.google.com/file/d/137fJzlpDIrk45ov-3n8QcVZ0vZnEZ5YE/view?usp=drive_link>

This request led us to reconsider our figures. Instead of adding a separate new figure, we revised our existing figures to provide the requested information and also added a pair of Tables which summarize key comparisons between experiment and simulation. The new versions of Fig. 8 and Fig. 10 now overlay model outputs with representative experimental/clinical data for epithelial thickness, turnover, and injury recovery. In addition, the new Table 2 summarizes, for four key corneal features, (i) the experimental/clinical ranges, (ii) the corresponding V-Cornea predictions under homeostasis or specific injury conditions, and (iii) whether each feature was used as a calibration target or treated as an emergent validation. We have added an extended version of Table 2 as Supplemental Table S9 for added clarity

(Line 711) To quantitatively link V-Cornea to human biology, we constrained the model using the limited number of relevant and available experimentally measured tissue-level features and then assessed additional emergent behaviors. Cell-level parameters (target cell sizes, mechanical constraints, EGF diffusion and decay) were bounded by literature values or conservative approximations (Supplementary Tables S8.1–S8.4). Within these bounds, we calibrated proliferation and sloughing rates to reproduce physiological epithelial turnover times of 7–14 days. We then evaluated independent emergent properties—epithelial thickness and limbal–central gradients, injury-specific healing times, and recurrent erosion-like instability after basement membrane damage—against clinical and experimental data (Figs. 3, 6–10). Table 2 summarizes this calibration–validation workflow; a more detailed version is provided in Supplementary Table S9.

(Line 722) Table 2 **Key corneal features used to calibrate and validate V-Cornea**.
A more detailed description is provided in Supplementary Table S9.

| **Feature** | **Experimental /  Clinical Value  (Range, with Refs)** | **V-Cornea Prediction  (This Study)** | **How Used in Model** |
| --- | --- | --- | --- |
| **Epithelial thickness and limbal–central gradient** | Human corneal epithelium ≈48–55 μm thick (31,83,103), 5–7 cell layers, limbal epithelium ~83 μm (84,104,105). | Stable 50–54 μm centrally; thicker (7–8 layers) at limbus  (Figs. 3 and 8). | **Emergent validation**: from cell growth, differentiation, and mechanical rules on physiologically bounded cells. |
| **Epithelial turnover time** | Complete renewal in  ~7–14 days (31,106–108). | Renewal of superficial layers in 7–14 days  (Figs. 6 and 7). | **Constrained calibration**: Turnover window guided tuning of proliferation and sloughing rates. |
| **Healing* time, slight/mild epithelial injury  (intact BM)** | Small injuries heal 1–3 days; larger epithelial injuries heal 3–5 days (87,109–111). | Closure in 3–5 days with recovery of thickness and cell counts  (Fig 9 and 10). | **Secondary constraint and validation**: Parameter sets failing clinical closure times rejected; model recovery matched clinical healing qualitatively. |
| **Healing* after BM damage (moderate injuries)** | BM disruption leads to slower closure and recurrent corneal erosion (RCE) over weeks-months (72,109–112). | No stable homeostasis by 20 days; recurrent epithelial breakdown like RCE (Fig 9 and 10). | **Qualitative emergent behavior**: Recurrent breakdown of epithelia emerges naturally, no specific parameters to confer instability. |

***Healing** refers to structural re-epithelialization and restoration of a continuous epithelial surface, as assessed by disappearance of fluorescein-stained defects and normalization of epithelial thickness in clinical imaging.

(Line 732)

***(Sup. Line 300) Table S9 - Expanded version of Table 2, providing detailed descriptions of key corneal features, their experimental/clinical values, V-Cornea predictions, and how each feature was used in model calibration and validation.***

| **Feature** | **Experimental / clinical value (range, with refs)** | **V-Cornea prediction (this study)** | **How used in model** |
| --- | --- | --- | --- |
| **Epithelial thickness and limbal–central gradient** | Human corneal epithelium ≈48–55 μm thick, 5–7 cell layers in the central cornea. (19–21) limbal epithelium ~83 μm μm.(22–24) | The model produces a stable thickness of 50–54 μm with 5–7 layers at homeostasis, and a thicker epithelium (up to 7–8 layers) over the limbal stem cell region, reproducing a limbal–central thickness difference (Figs. 3 and 8). | Emergent validation. Cell sizes and mechanical parameters were set within physiologically plausible ranges based on in vitro measurements and corneal imaging (Supplementary Tables S8.1–S8.4), but neither absolute epithelial thickness nor a limbal–central difference was directly imposed. Both features emerge from the interaction of growth, differentiation, and mechanical rules acting on these physiologically bounded cells. |
| **Epithelial turnover time** | Estimates based on in vivo labeling and imaging studies suggest full corneal epithelial turnover in roughly 7–10 days, with some reports up to 14 days; direct quantitative data are limited, but multiple kinetic studies are consistent with this range (20,25–27) | The model shows ~7–14 days for complete renewal of superficial layers in the central and peripheral epithelium. | Constrained calibration. We used the 7–14 day turnover window as a primary calibration target when tuning proliferation rates and superficial sloughing rate (Tables S8.1–S8.4). Only parameter sets that produced full renewal within this window and preserved realistic epithelial thickness and limbal–central gradients (Row 1) were accepted, ensuring that fitted parameters remained within physiologically reasonable ranges*.* |
| **Healing time, slight/mild epithelial injury (intact BM)** | Small/superficial corneal abrasions usually heal in 1–3 days; and larger but still epithelial-only abrasions heal within about 3–5 days (28–31). Here ‘healing’ refers to structural re-epithelialization and restoration of a continuous epithelial surface, as assessed by disappearance of fluorescein-stained defects and normalization of epithelial thickness in clinical imaging. | Slight and mild injuries confined to the epithelium close completely in ~3–5 days, with re-establishment of a continuous epithelial surface and return of thickness and cell counts to pre-injury baseline (Fig. 9–10, top/middle rows). | Secondary constraint + validation. During parameter screening, we rejected parameter sets for which slight/mild epithelial-only injuries failed to close within the 1–5 day clinical window (Row 3, experimental column). However, we did not fit an exact time course. After fixing parameters based on homeostasis and turnover (Rows 1–2), we confirmed that the resulting wound-closure dynamics, recovery of epithelial thickness and cell counts, and near-exponential decrease in defect area qualitatively match clinical observations of epithelial sheet migration and area closure |
| **Healing after basement membrane damage (moderate injuries)** | When the epithelial basement membrane is disrupted, re-epithelialization and restoration of stable epithelial adhesion are slower and prone to recurrent erosions; clinically, structurally closed epithelia may repeatedly break down for weeks to months, resulting in recurrent corneal erosion (RCE), where structurally closed epithelia reopen under minor stress. (29–33) | Moderate injuries that destroy EpBM do not fully return to stable homeostasis over 20 days. After initial closure, the model exhibits repeated episodes of epithelial detachment and loss of superficial/wing layers, with incomplete recovery of cell numbers and thickness—behavior qualitatively similar to clinical RCE. | Qualitative emergent behavior. Recurrent breakdown of the epithelium arises naturally from impaired basement membrane adhesion. For example, we did not add any extra rules (e.g., special “RCE” states or injury-depth-dependent sloughing); the same parameter set calibrated for homeostasis and epithelial-only injuries (Rows 1–3) automatically produces RCE-like instability when EpBM is removed. |

**2) Add a detailed discussion on the limitations of the 2D modeling compared to a full 3D model (how do scaling relations change? how do injury progression changes? etc.)**

We have substantially expanded Section 2.2 (“Simulation Domain and Spatial Framework”) to clarify how our 2D radial cross-section relates to the 3D cornea and to discuss the resulting limitations. Specifically, we now (i) describe the geometric approximation of a curved 3D shell by a flat 2D domain and quantify that the curvature-induced geometric distortion over the 0.4 mm simulated radial extent is small (on the order of a single lattice spacing); (ii) explain that cell numbers, wound areas, and chemical loads in the model should be interpreted as densities per unit circumferential length, and that absolute 3D quantities scale with the implicit circumferential extent of the modeled slice; and (iii) discuss how dimensional reduction alters local mechanics—replacing 3D contact areas with 2D contact lengths, increasing effective crowding, and preventing out-of-plane rearrangements.

We also now explicitly distinguish which aspects of injury dynamics are well captured by the 2D formulation and which require a 3D or surface (r, θ) representation. In the revised text, we state that our simulations correspond to the radial component of wound closure and depth-dependent injury progression, calibrated to match experimental epithelial thickness, turnover times, and healing times for epithelial-only injuries. In contrast, phenomena that depend on circumferential heterogeneity—such as sectoral limbal stem cell loss, circumferential conjunctivalisation, or spatially complex injury geometries—are outside the scope of the current 2D implementation and would require a 3D extension of V-Cornea. We believe this revised section now makes the scaling assumptions and limitations of the 2D model explicit and directly addresses the reviewer’s request.

## **2.2 Simulation Domain and Spatial Framework**

(Line 326) Our model represents a two-dimensional (2D) radial sagittal section of the corneal limbus and peripheral cornea. The simulation domain is implemented as a discrete square lattice of 200×90 voxels [Fig. 3], where each voxel corresponds to a 2×2 μm area (4 µm²), resulting in a total simulated region of 400×180 µm. Within this domain, the limbal region spans approximately 80 µm in width, while the peripheral corneal region extends a further ~320 µm toward the central cornea. We restrict the domain to the limbus and adjacent peripheral cornea rather than the entire cornea for computational efficiency and because this region contains the limbal stem cell niche and captures the key epithelial dynamics of interest. The modeled epithelial thickness is ~50–52 μm, corresponding to ~4–8 cell layers, which falls within the range of central epithelial thickness reported for healthy human corneas by SD-OCT and AS-OCT imaging (≈48–55 μm) (83). Limbal epithelial thickness measurements in normal eyes are typically around 80–85 μm (84), substantially thicker than the central epithelium. Our simulated limbal region is correspondingly thicker than the central epithelium, consistent with this limbal–central gradient observed in vivo. We modeled epithelial cell diameters ranging from 10–30 μm (85) and implemented a simulated tear film layer of 5–10 μm to reflect physiological conditions.

Fig 3. **Simulated corneal epithelium structure compared to histology.** (A) Histological cross-section showing stratified layers and cell density. (B) V-Cornea CompuCell3D rendering ( voxels). The model represents the Stroma (lilac-purple), Bowman’s layer/EpBM (pink/magenta), Limbal Stem Cells (rose-red), Basal (peach-orange), Wing (blue), and Superficial cells (cyan) topped by Tear film (green). The medium (black) represents air. Panel A adapted from Shortt et al. (84)

Geometrically, the in vivo cornea is part of a curved 3D shell with an average anterior radius of curvature of approximately 7.5–8.0 mm (86). Our 2D model approximates this shell by a flat rectangular domain along one meridian. Because the simulated radial extent (400 μm) is small compared with the corneal radius, the curvature-induced distortion is modest: a 400 μm chord on a sphere of radius ~7.8–8.0 mm the implied “thickness” of the radial slice changes by only a few percent across the domain. Thus, over the spatial scales we consider, flattening the curved shell introduces only small errors in path lengths and local interface geometry for migrating cells, relative to biological variability in epithelial thickness, cell size, and motility.

A more important consequence of the 2D representation is how it relates to the full 3D tissue. Each point in our 2D radial slice corresponds, in vivo, to a ring of tissue that extends around the corneal circumference at that radius. Quantities such as cell number, wound area, and chemical load in the model should therefore be interpreted as *densities per unit circumferential length* (analogous to “cells per millimeter of corneal circumference”) rather than absolute totals for the entire cornea. In a 3D eye with a roughly circular epithelial defect, the true wound area at radius scales with the circumferential length, while our 2D slice describes how far the epithelial front has advanced along one radial line. Provided limbal function is approximately uniform around the circumference, the radial healing kinetics in our slice (front position vs. time and depth) are expected to match the local contribution to global wound closure, even though the model does not explicitly track the full plan-view wound shape.

This interpretation is consistent with clinical imaging of large epithelial abrasions. Dua and Forrester (87) showed that re-epithelialization in patients follows a reproducible pattern: three to six convex epithelial fronts develop along the wound circumference and progress toward the center, neighboring fronts meet to form polygonal contact geometries, and the final closure often exhibits a characteristic Y-shaped contact line. The area of the epithelial defect decreases approximately exponentially with time, indicating an almost constant rate of epithelial migration along the wound edge. In this regime, each radial meridian across the defect effectively experiences a locally advancing front with similar speed. Our 2D radial model is designed to capture exactly this radial component of healing—how an epithelial front advances with depth and radial position—rather than the global plan-view geometry of multiple fronts and their circumferential interactions.

The dimensional reduction from 3D to 2D also affects mechanics and cell rearrangements. In the real cornea, epithelial cells and the underlying extracellular matrix can deform and rearrange in three dimensions, including circumferentially and out of the sagittal plane. In contrast, cells in our 2D cross-section are confined to a plane: contact interfaces are represented as line segments rather than surface areas, forces are effectively projected into that plane, and cells cannot “move around” each other by stepping into the third dimension. This constraint tends to increase local crowding and jamming at a given cell density and alters how mechanical stresses percolate through the tissue compared to a 3D model. As a result, we do not interpret parameters such as contact energies or motility coefficients as direct estimates of 3D mechanical quantities; instead, we treat them phenomenologically and calibrate them so that the model reproduces population-level observables such as epithelial thickness profiles, turnover times, clonal patterns, and wound closure times.

These scaling and mechanical considerations also define which questions the current 2D framework can, and cannot, address. V-Cornea is well suited to studying local depth- and radius-dependent behaviors: maintenance of homeostasis, differences between slight (superficial) and mild (full-epithelium) injuries, the consequences of basement membrane damage, and the associated healing time courses when limbal stem cell supply is intact and approximately symmetric. In contrast, phenomena that are fundamentally circumferential or sectoral—such as the emergence of multiple discrete fronts and Y-shaped contact lines in plan view, asymmetric healing when only part of the limbus is functional, or progressive conjunctivalisation creeping across the cornea in limbal stem cell deficiency—lie outside the scope of a single 2D radial slice. Addressing those questions would require at least a surface model defined over radius and angle (r,θ), or a full 3D implementation of the corneal epithelium and conjunctiva.

Within this interpretation, the modest geometric distortions introduced by flattening the cornea and the enhanced crowding inherent to 2D are absorbed into the calibrated model parameters. Consequently, even though individual cell–cell and cell–matrix interactions in the simulation differ quantitatively from those in a full 3D tissue, the emergent tissue-level behaviors we focus on—homeostatic epithelial turnover, classification of injuries by depth, and the contrasting recovery dynamics of epithelial-only versus basement-membrane–disrupting injuries—remain representative of biological observations. The spatial mapping (2 μm per lattice site) and temporal mapping (1 Monte Carlo step ≈ 6 minutes of biological time) provide a direct correspondence between simulation outputs and experimental or clinical measurements of epithelial thickness and wound closure dynamics.

**3) It is not easy to get a clear, full picture of the equations and the biological numbers (and their justification) that are in many tables and sub-sections. Add a clear table with all the numbers with units and their corresponding equations.**

We thank the reviewer for this suggestion. We agree that consolidating the mathematical formulations and biological parameters improves the transparency and reproducibility of the model. We have added a new table (Table 1) that lists key parameters with associated values, units, and biological justification/references.

(Line 574) Table 1 **Summary of core model components and rules.**
Full mathematical details and parameter values are provided in Supplementary Information S1–S5

| **Model**  **quantity / rule** | **Value**  **(simulation units)** | **Approximate biological**  **value / target** | **Role in model / interpretation** |
| --- | --- | --- | --- |
| **Spatiotemporal scales** | | | |
| **Lattice Unit Edge Length** | 1 voxel unit | 2 μm | Spatial resolution of the epithelial cross-section and wound geometry. |
| **Lattice Unit Area** | $1 \mathrm{voxel}^{2}$ | $4.0 \mu m^{2}$ | Area per lattice site directly derived from edge length. |
| **Time Step** | $1 MCS$ | 6.0 min | Decision based on computing power and resolution of cellular behavior like movement and division. |
| **Simulation Domain** | $200\times$ $90 voxels$ | $400\times$ $180 \mu m$ | Represents limbus + peripheral cornea. (Fig. 3) while computationally tractable. |
| **Growth and turnover** | | | |
| **Growth rate of proliferating cells** | Rate-limiting function (Eq. 3) | $\delta_{i}$ set so that, under high EGF and low crowding, cells can double volume in  ≈ 8 h (29) | Combines biochemical (EGF) and mechanical (density/pressure) cues; defines maximum proliferative capacity under optimal conditions. |
| **EGF-dependent growth rate of cells** | Hill function of local EGF (Eq. 1) | Increase in growth when EGF exceeds a half-max level | Represents MAPK/ERK-like ultrasensitive growth-factor response and noise-filtering behavior (54,88). |
| **Half-Max EGF concentration for EGF-dependent growth rate**  **(Stem) / (Basal)** | $k_{m,EGF,STEM}=3.5$  $arbritrary units$/  $k_{m,EGF,BASAL}=7.0$  $arbritrary units$ | Local cell response at which growth rate is increased by ~50% | Calibrated so epithelial thickness and limbal–peripheral density gradients remain physiological while maintaining 7–14 day renewal (3). |
| **Density inhibition rate of proliferating cells** | Hill function of effective pressure  (Eq. 2) | Steep decline of growth with increasing crowding | Represents contact inhibition of proliferation in crowded epithelia (30). |
| **Half-Max pressure for contact inhibition of cell growth** | $k_{m, density}= 125.0$ $arbritrary units$ | Local pressure at which growth is reduced by ~50% | Fitted density/crowding level where proliferation becomes strongly inhibited at steady state. |
| **Superficial cell sloughing rate** | Stochastic Rule/Process  (Eq. 8) | ≈ 1.75 days per layer → 7–14 days total renewal | Probabilistic removal of superficial cells at the tear interface; matches reported corneal epithelial turnover times (90,95). |
| **Differentiation and EGF dynamics** | | | |
| **Differentiation rules (LESC→Basal, Basal→Wing, Wing→Superficial)** | Logical rules based on neighbor set $N$ and EpBM contact  (Eqs. 4–6) | Immediate state transitions once spatial conditions are met | Implement deterministic, spatially triggered differentiation cascade from limbal niche to apical surface (45,48,49,57,58). |
| **EGF reaction–diffusion dynamics** | Global diffusion rate =$186.0$ ${\mathrm{voxe}l^{2}}/\mathrm{MCS}$  Global decay = 0.5 $\mathrm{MC}S^{-1}$ (Eq. 9) | $2.1\times{10}^{-8}$ $cm^{2}/s$ (93)  ~42-114 min  (94) | Captures EGF gradients driven by a constant Tear Film source (34,35) and cell-type–dependent diffusion (50), regulating proliferation and migration during homeostasis and injury. |

Reviewer #2: This manuscript presents an agent-based model on corneal epithelial development dynamics, which combines cell movement driven by intercellular forces and cell differentiation governed by a set of simplified biological rules. The model simulation was implemented through the CompuCell3D framework. The model reproduces corneal epithelial homeostasis and responses to mild injuries. The model predictions for moderate injuries that harms the basement membrane recapitulates recurrent corneal erosions, although further incorporation of cellular dynamics beyond the epithelium is necessary for accurate quantitative prediction. The model provides a prototype predictive tool for biomedical and clinical studies of corneal injuries, which can be extended in the future with more cellular mechanisms incorporated. The manuscript is written with high clarity.

Major comments:

**1. Is there any physiological or modeling reason behind the Hill exponent of 4 in the dependence of proliferation rates of stem cell and basal cell on EGF and cell density?**

We thank the reviewer for this thoughtful questionregarding the Hill exponent (n=4). While this term serves as a phenomenological parameter in our ODE system, the choice of n ~4 is grounded in the specific architecture of epithelial signaling and contact inhibition:

**Growth Factor Ultrasensitivity:** The proliferative response to EGF is mediated largely by the MAPK/ERK cascade. Huang and Ferrell (1996) demonstrated that the multi-step phosphorylation in this cascade converts graded inputs into switch-like outputs, generating an apparent Hill coefficient of ~4.9 (Huang & Ferrell, *PNAS*, 1996). This creates a necessary noise-filtering mechanism, ensuring that low levels of growth factor do not trigger aberrant proliferation.

**Density-Dependent Inhibition:** Similarly, the cessation of division due to crowding (contact inhibition) follows a sharp transition. Puliafito et al. (2012) quantified the relationship between local cell density and mitotic rate in epithelial sheets, finding that the data explicitly fits a Hill function with an exponent of m ~4 (Puliafito et al., *PNAS*, 2012).

**Model Performance:** These biological findings align with our simulation results. In our preliminary testing, lower coefficients (e.g., n=1 or 2) produced "leaky" proliferation where cells continued to divide beyond physiological limits. By utilizing n=4, the model reproduces the distinct “switch-like” proliferative states required to satisfy observed biological constraints: (i) 7–14 day epithelial turnover, (ii) stable stratification of 5–7 layers, and (iii) accurate injury-healing timelines.

To clarify that in the main text we added on section 2.4.1 the following paragraph:

(Line 448) In both Hill functions we use an exponent n=4. This value is not meant to represent the stoichiometry of a single molecular interaction, but rather an effective ultrasensitive switch between proliferative and quiescent states. Multi-step kinase cascades such as MAPK/ERK, which mediate growth-factor responses, produce stimulus–response curves with apparent Hill coefficients on the order of 4–5 (Huang & Ferrell, PNAS, 1996), reflecting strong ultrasensitivity and noise filtering in the proliferation decision. Likewise, quantitative measurements of contact inhibition in epithelial monolayers show that the decline of mitotic rate with increasing local density is well described by a steep Hill function, with exponents near 4 (Puliafito et al., PNAS, 2012). In preliminary simulations, lower exponents (e.g. n=1–2) produced overly graded, “leaky” proliferation and failed to maintain realistic epithelial thickness, turnover times, and injury-healing dynamics, whereas n=4 allowed us to satisfy these constraints simultaneously.

Here is the comparison between the different powers

**2. Where are the various layers in Fig.5? I presume the red region before injury and after recovery correspond to the tear film, as EGF concentration was highest there? What confuses me is the EGF concentration beyond that tear layer. Shouldn’t there be nothing beyond the tear? If it is a numeric convenience to assume diffusion of EGF beyond the tear film, would this artificial diffusion cause underestimation or some sort of distortion of the EGF level in the cornea?**

We appreciate the reviewer's close examination of the EGF field in Figure 5. To clarify the spatial arrangement: the red region corresponds to the Tear film, the gradient below it represents the corneal tissue (Superficial to Basal layers), and the region above the Tear film represents the Medium (air).

Regarding the EGF concentration in the "air": In our simulation, the Tear film is modeled as a constant concentration source of EGF, rather than a finite pool of diffusing molecules. This fixed connotation source comes close to physiological reality where the tear film and the EGF it contains is continuously replenished by the lacrimal glands, so there is very little change of EGF level in time or across the diameter of the cornea. Thus, while the figures would look more aesthetically pleasing if we inhibited diffusion into the “air”, the diffusion of EGF into the “air” actually has no effect on the diffusion of EGF into the tissue.

Because the Tear film acts as a constant source (maintained at normalized concentration = 1.0), the diffusive flux into the Medium (air) does not deplete the concentration available at the epithelial interface. The boundary condition experienced by the corneal tissue remains constant, ensuring there is no underestimation or distortion of the EGF levels within the tissue. The gradient visible in the Medium is a visualization artifact of the simulation grid boundaries (which act as a sink at y=Ly) but has no mathematical impact on the tissue dynamics.

We have updated the figure caption to better identify the layers and added a clarification in Section 2.5 regarding the constant source implementation.

(Line 543)…This simplified representation allows us to capture essential regulatory dynamics without the computational complexity of modeling each signaling pathway individually. The tear film serves as a constant EGF source at the epithelial surface, modeled as a region of fixed concentration to represent continuous lacrimal replenishment. The EGF concentration field evolves according to the equation:

Minor comments:

**1. Ln. 114-119: This sentence is really difficult to read. To improve clarity, it would be better to break it into two sentences.**

Thanks for your careful reading and suggestion, we modified the last paragraph of section 1.1 the lines you mentioned to:

(Ln. 132)... Our Virtual Cornea (V-Cornea) combines mechanistic rules for individual cell behaviors with a simplified representation of chemical signaling and tissue architecture. This design yields a single platform that unifies cellular, mechanical, and biochemical aspects for studying corneal injury and recovery dynamics.

**2. Ln. 257: “While … “ According to the meaning of the sentence, “As/Since/Because” may be more appropriate for the first word?**

Thanks for this suggestion. We corrected it to (Ln. 274)" ... Since the stroma is minimally …”

**3. The “if” conditions given in Eqs. (4) and (6) contain math notations that I don’t understand.**

Thank you so much for catching that! We apologize for the confusion caused by the mathematical notation. We have simplified the equations to clearer logical statements and expanded the text descriptions to explain exactly what spatial conditions trigger differentiation. Here is the new version:

## (Ln. 473) **2.4.3 Differentiation**

Cell differentiation is implemented as a rule-based process triggered by spatial relationships. **LESC to Basal** transition occurs when an LESC loses contact with the limbal EpBM [Fig. 4 - first rule] (45,57), represented as

where *N* is the set of cell types neighboring the cell being evaluated, and the symbol indicates that the **LIMB** cell type is *not* present in that neighborhood.

**Basal to Wing** transition is triggered when contact with the basement membrane diminishes (48) below a defined threshold:

where is the number of pixels in contact with the basement membrane, and pixels is the minimal contact area required to maintain basal phenotype.

**Wing to Superficial** transition occurs when wing cells reach the uppermost region (49,58) To differentiate, the cell must contact the Tear film **and** must have completely lost contact with the lower layers (Basal, Stem, or Basement Membrane):

In this equation, requires contact with the tear film, while the second condition specifies that the intersection () between the cell's neighbors () and the lower-layer cell types must be empty (). These transitions are deterministic and occur immediately once the conditions are met.

**4. What is the reason that the number of layers of wing cells is highest right above the stem cells and become largely constant towards the central cornea?**

This reminded us to include the reference for the differences in thickness between the limbus and the rest of the cornea. We made the clarification of the differences and also included a visual reference to the plots for Fig 8 and Fig 10.

(Ln. 326) Our model represents a two-dimensional (2D) radial sagittal section of the corneal limbus and peripheral cornea. The simulation domain is implemented as a discrete square lattice of 200×90 voxels [Fig. 3], where each voxel corresponds to a 2×2 μm area (4 µm²), resulting in a total simulated region of 400×180 µm. Within this domain, the limbal region spans approximately 80 µm in width, while the peripheral corneal region extends a further ~320 µm toward the central cornea. We restrict the domain to the limbus and adjacent peripheral cornea rather than the entire cornea for computational efficiency and because this region contains the limbal stem cell niche and captures the key epithelial dynamics of interest. The modeled epithelial thickness is ~50–52 μm, corresponding to ~4–8 cell layers, which falls within the range of central epithelial thickness reported for healthy human corneas by SD-OCT and AS-OCT imaging (≈48–55 μm) (83). Limbal epithelial thickness measurements in normal eyes are typically around 80–85 μm (84), substantially thicker than the central epithelium. Our simulated limbal region is correspondingly thicker than the central epithelium, consistent with this limbal–central gradient observed in vivo. We modeled epithelial cell diameters ranging from 10–30 μm (85) and implemented a simulated tear film layer of 5–10 μm to reflect physiological conditions.

84 -> Francoz M, Karamoko I, Baudouin C, Labbé A. Ocular Surface Epithelial Thickness Evaluation with Spectral-Domain Optical Coherence Tomography. Invest Ophthalmol Vis Sci. 2011 Nov 24;52(12):9116–23.

**—------------------------------------------------------------------------------------------------------**

Have the authors made all data and (if applicable) computational code underlying the findings in their manuscript fully available?

The PLOS Data policy requires authors to make all data and code underlying the findings described in their manuscript fully available without restriction, with rare exception (please refer to the Data Availability Statement in the manuscript PDF file). The data and code should be provided as part of the manuscript or its supporting information, or deposited to a public repository. For example, in addition to summary statistics, the data points behind means, medians and variance measures should be available. If there are restrictions on publicly sharing data or code —e.g. participant privacy or use of data from a third party—those must be specified.

Reviewer #1: None

Reviewer #2: Yes

PLOS authors have the option to publish the peer review history of their article (what does this mean?). If published, this will include your full peer review and any attached files.

If you choose “no”, your identity will remain anonymous but your review may still be made public.

Do you want your identity to be public for this peer review? For information about this choice, including consent withdrawal, please see our Privacy Policy.

Reviewer #1: No

Reviewer #2: No

[NOTE: If reviewer comments were submitted as an attachment file, they will be attached to this email and accessible via the submission site. Please log into your account, locate the manuscript record, and check for the action link "View Attachments". If this link does not appear, there are no attachment files.]
